# Inflammation and JNK's Role in Niacin-GPR109A Diminished Flushed Effect in Microglial and Neuronal Cells With Relevance to Schizophrenia

**DOI:** 10.3389/fpsyt.2021.771144

**Published:** 2021-11-30

**Authors:** Sabrina H. Ansarey

**Affiliations:** Department of Neuroscience and Psychology, University of Glasgow, Glasgow, United Kingdom

**Keywords:** diminished GPR109A-flushed effect, niacin, microglia, JNK treatment, schizophrenia, c-Jun N-terminal kinase (JNK) pathway, neuron

## Abstract

Schizophrenia is a neuropsychiatric illness with no single definitive aetiology, making its treatment difficult. Antipsychotics are not fully effective because they treat psychosis rather than the cognitive or negative symptoms. Antipsychotics fail to alleviate symptoms when patients enter the chronic stage of illness. Topical application of niacin showed diminished skin flush in the majority of patients with schizophrenia compared to the general population who showed flushing. The niacin skin flush test is useful for identifying patients with schizophrenia at their ultra-high-risk stage, and understanding this pathology may introduce an effective treatment. This review aims to understand the pathology behind the diminished skin flush response, while linking it back to neurons and microglia. First, it suggests that there are altered proteins in the GPR109A-COX-prostaglandin pathway, inflammatory imbalance, and kinase signalling pathway, c-Jun N-terminal kinase (JNK), which are associated with diminished flush. Second, genes from the GPR109A-COX-prostaglandin pathway were matched against the 128-loci genome wide association study (GWAS) for schizophrenia using GeneCards, suggesting that G-coupled receptor-109A (GPR109A) may have a genetic mutation, resulting in diminished flush. This review also suggests that there may be increased pro-inflammatory mediators in the GPR109A-COX-prostaglandin pathway, which contributes to the diminished flush pathology. Increased levels of pro-inflammatory markers may induce microglial-activated neuronal death. Lastly, this review explores the role of JNK on pro-inflammatory mediators, proteins in the GPR109A-COX-prostaglandin pathway, microglial activation, and neuronal death. Inhibiting JNK may reverse the changes observed in the diminished flush response, which might make it a good therapeutic target.

**Graphical Abstract:**
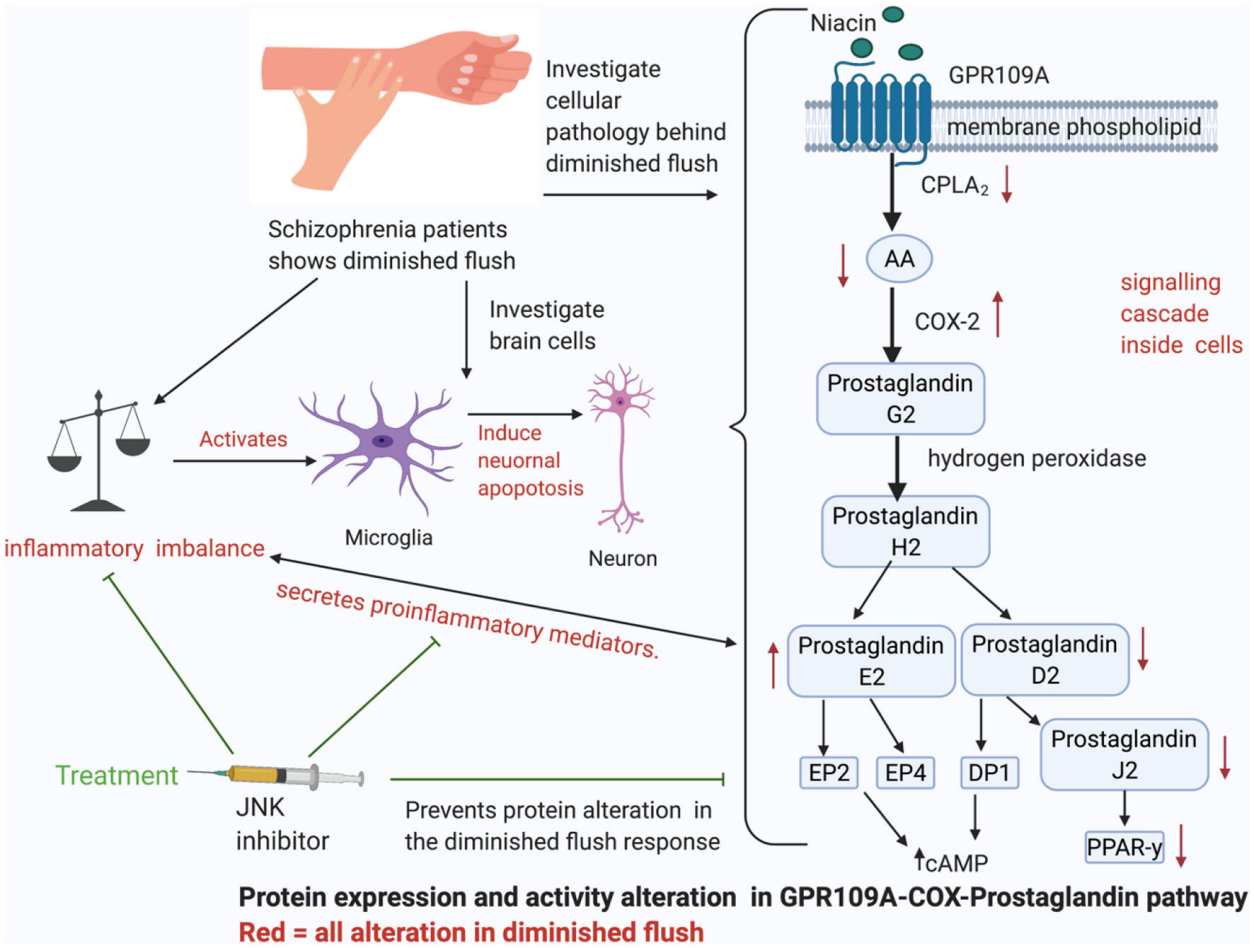
The cellular pathway behind diminished skin flush. The GPR109A-COX-prostaglandin pathways shows how niacin binds to G-coupled receptor-109A (GPR109A) on the cell membrane of leukocytes, and stimulates a cascade of signals, which activates enzymes to release vasodilators, prostaglandin D_2_ and E_2_. This pathway induces pro-inflammatory mediators, which can then activate M1 microglia to induce neuronal apoptosis. JNK inhibitors have been observed to act as an anti-inflammatory agent, reduce excessive pro-inflammatory mediators, and prevent microglial induced neuronal apoptosis.

## Introduction

Our society has neglected satisfactory categorisation of mental illness for over 2,000 years (1). In the past, schizophrenia had failed to be defined and understood as its own entity (2). The term schizophrenia was coined by Blueler (3, 4). Blueler and Kraepelin described the symptoms and aetiology of the illness (5). Schizophrenia is diagnosed by its symptoms, where positive symptoms include hallucinations, delusions, disorganised thoughts, and speech; negative symptoms include anhedonia, apathy, and social withdrawal; and cognitive symptoms include inattention, impaired working memory, and dysfunctional executive functions, which affect thoughts, intelligence, and ability to plan. Most individuals diagnosed with schizophrenia undergo a prodromal stage before full-blown psychotic symptoms appear, where individuals experience changes in both cognition and behavioural function (6, 7). The early onset of symptoms usually occurs between the ages of 14 to 29 (4); therefore, identifying the ultra-high-risk population is crucial for initiating early intervention.

Schizophrenia represents one percent of the global population and remains one of the top 25 leading disability worldwide (8). The World Health Organisation estimated that the direct cost for schizophrenia ranges from US$94 million to $102 billion (9). However, the substantial burden of the illness has been linked to its early onset and incurable nature with persistent symptoms (10). Heterogeneous illnesses have other problems, where a majority of research focuses on the altered neurotransmitter function of schizophrenia, typically dopamine or glutamate, in which treatments associated with this paradigm (currently dopamine antagonists) fail to alleviate negative and cognitive symptoms in 30–60% of the patients (11–14). Current antipsychotics increase the risk of other comorbidities associated with the heart (15) or diabetes (16).

Alternative approaches should be considered when treating this complex disorder. Both Kraepelin and Bluer identified that the aetiology of schizophrenia is a consequence of gene-environment interactions (5). Dr. Hoffer proposed megavitamin B3 therapy, in which niacin (vitamin B3) intake over time reduces symptoms of schizophrenia (17). The general population exposed to niacin showed skin flush as a side effect (18), whereas, niacin exposure in the majority of schizophrenia patients showed diminished skin flush (19–22). The diminished flush response serves as an endophenotype and separates patients with schizophrenia from other mood disorders such as depression (23), bipolar disorder (24, 25), and social phobia (26).

**Figure 1:**
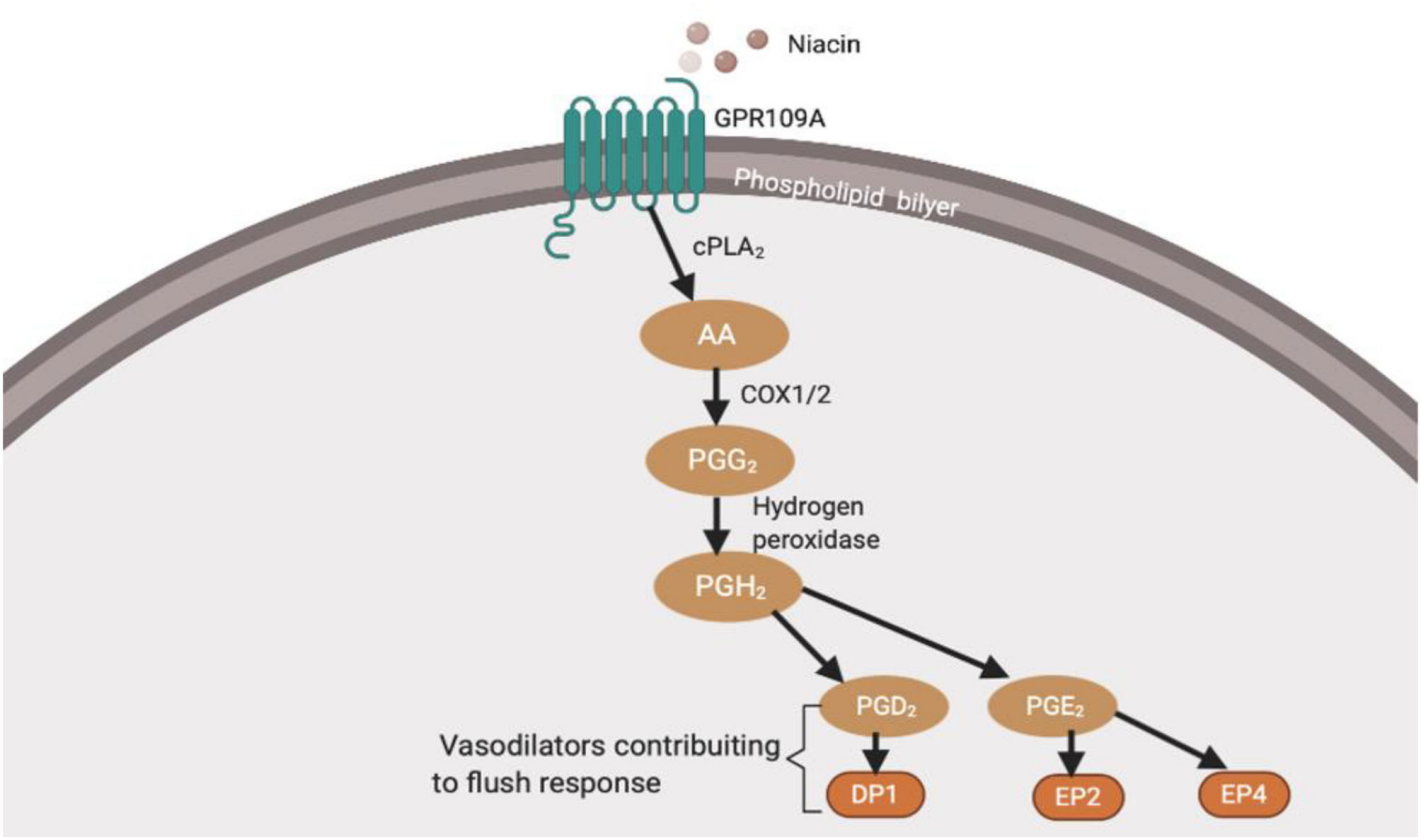
Niacin binds and activates GPR109A receptors on dermal cells, epidermal langerhans cells, and adipose macrophages (27). In langerhans cells, the activated receptor releases intracellular calcium (Ca^2+^) (28), which triggers phospholipase A_2_ (PLA_2_) to catalyse the breakdown of membrane phospholipid to arachidonic acid (AA). Available AA is converted to eicosanoid, prostaglandin G_2_ (PGG_2_) and prostaglandin H_2_ (PGH_2_) *via* COX1/2 and hydrogen peroxidase respectively. PGH_2_ is converted to various prostaglandins, prostacyclins, and leukotrienes. However, because this thesis focuses on the diminished flush observed in schizophrenia, I will focus on prostaglandins (29). Mediators involved in the cutaneous flushing are vasodilators, prostaglandin D2 (PGD_2_), and E2 (PDE_2_), which activates prostaglandin D_2_ receptor 1 (DP_1_) and prostaglandin E_2/4_ receptor (EP_2/4_), respectively (25). Moreover, these are biochemical alteration which may be partially inherited (30, 31). However, the pathophysiology of the attenuated flush response is not fully understood.

Prostaglandins in the cyclooxygenase (COX) pathway have been connected to flushing (Figure 1). However, it is unclear how these prostaglandins are deactivated or reduced in patients with schizophrenia. Other factors that have been thought to influence diminished flush include smoking (32, 33), alcohol consumption, caffeine intake, use of medicine (34), and altered chemical mediators such as nitric oxide (NO) (35, 36), histamine, and adrenaline (37). The aberrant immune response observed in these patients may be related to a diminished flush response (38–44). While current studies link the diminished flush in peripheral immune cells, this review aims to investigate possible links between diminished niacin-GPR109A flush responses mediated via the GPR109A-COX-prostaglandin pathway in microglia and neuronal cells. This study aimed to investigate the link between altered protein expression or activity in the GPR109A-COX-prostaglandin pathway with associated inflammatory mediators and the c-Jun N-terminal kinase (JNK) signalling pathway in patients with schizophrenia. Furthermore, GeneCards were used to manually identify chromosome numbers of genes from the GPR109A-COX-prostaglandin pathway with a 128-loci GWAS for schizophrenia (45).

## Neuroinflammation

**Figure 2:**
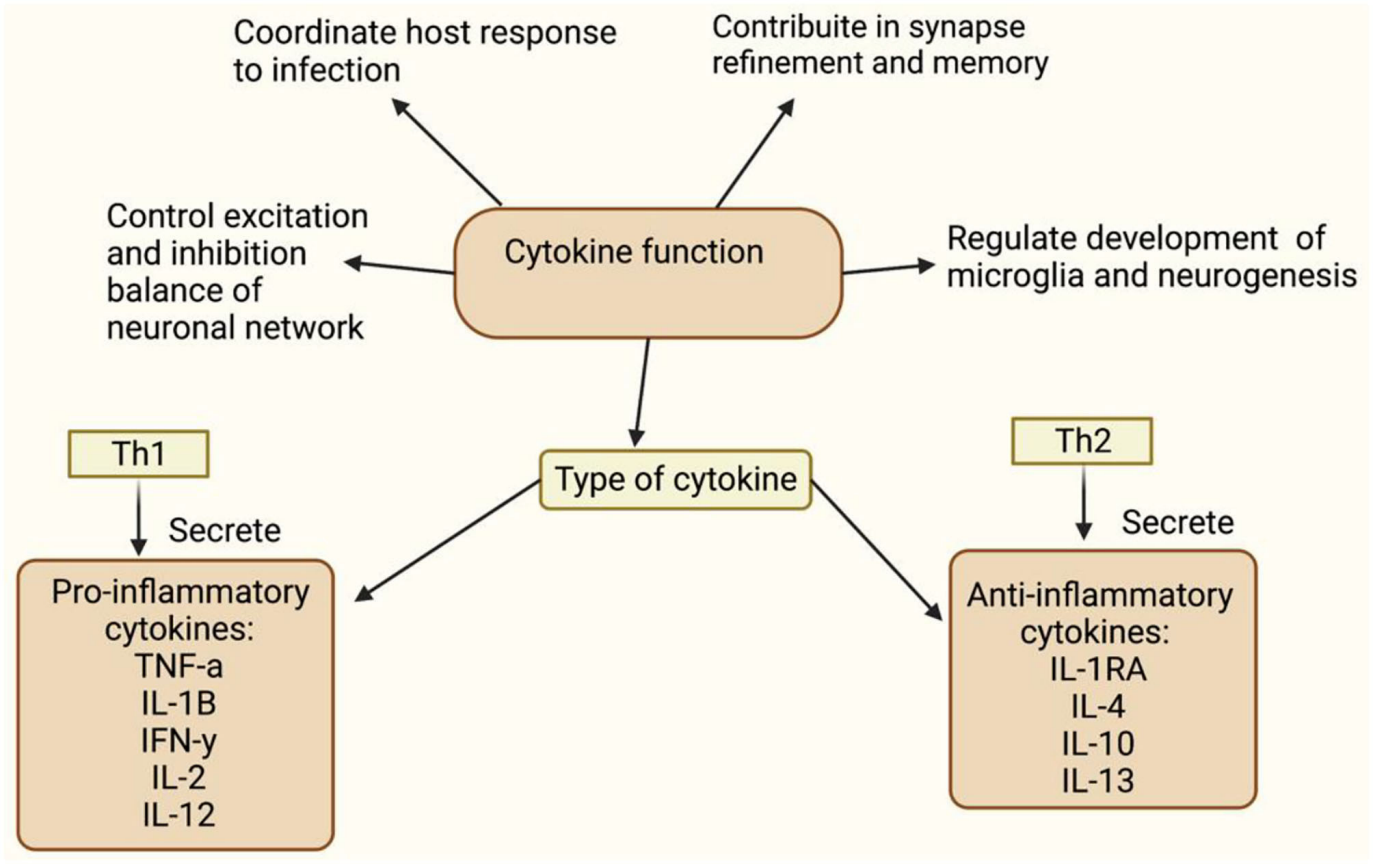
This figure shows function of cytokine (48–51). Tumour necrosis factors-a (TNF-a), Interleukin-1B (IL-1B), Interferon necrosis- y (IFN-y), Interleukin-2 (IL-2), Interleukin-12 (IL-12), Intereukin-4 (IL-4), Interleukin-10 (IL-10), Interleukin-13 (IL-13). Understanding the cytokine function will help to understand inflammation's involvement in diminished flush response and its role in activating microglia, respectively. Elevated pro-inflammatory levels, IL-1B and IL-6, and decreased anti-inflammatory levels have been observed in schizophrenia (52–54). Raison et al. (55) reported increased IL-1B and TNF-a observed in negative and cognitive symptoms of schizophrenia. Goldsmith et al. (56) and Wang and Miller (57) meta-analysis showed consistent upregulated pro-inflammatory cytokine, but variation in anti-inflammatory cytokine levels. Variation in anti-inflammatory markers, may be due to confounding factors, such as stage of illness, gender, age and medication status. Miller et al. (40) and Khandaker et al. (58) showed alternated cytokine levels in different stages of illness, which includes early-onset childhood, acute and relapse phase.

**Figure 3:**
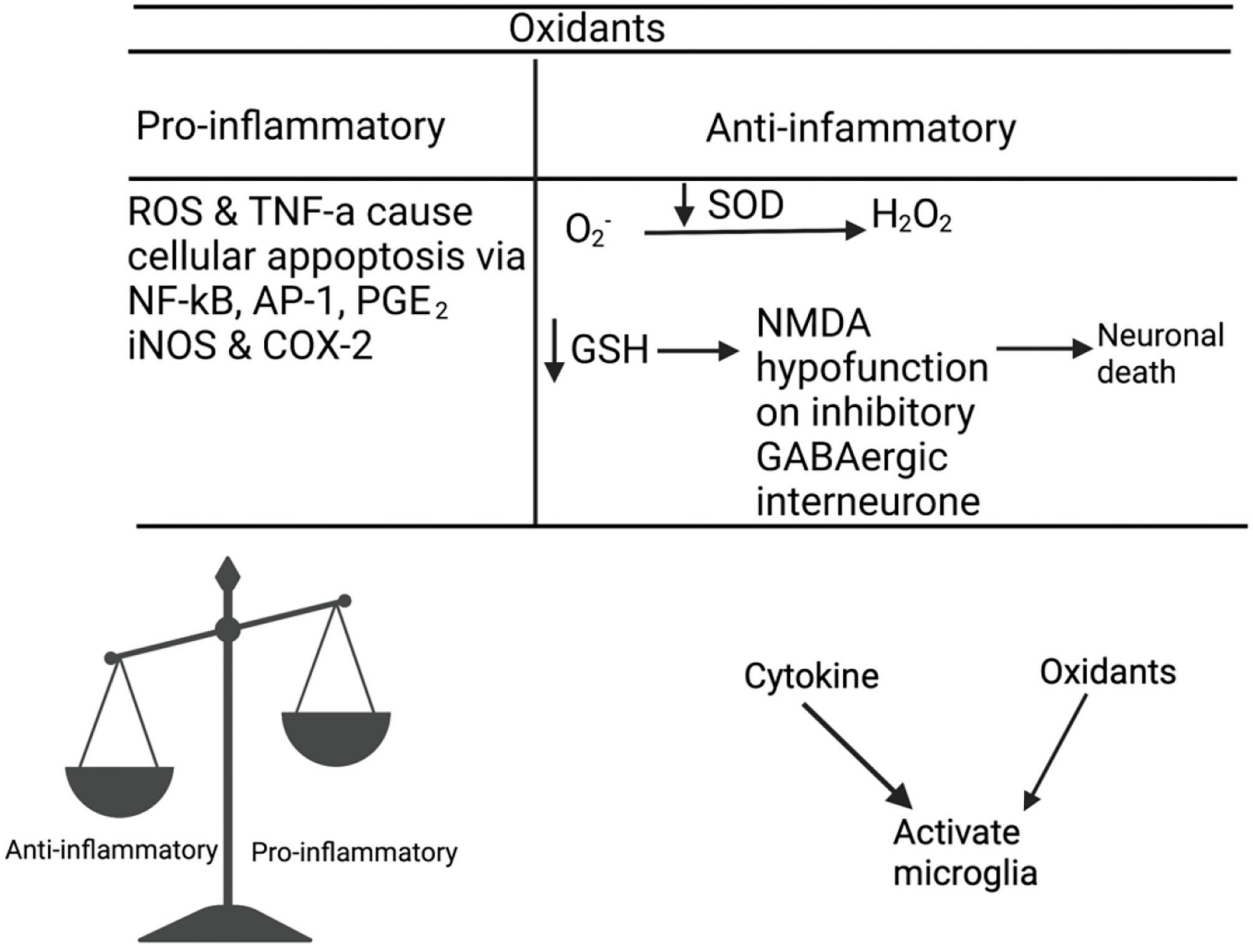
Matsubara and Ziff (59) and Meier et al. (60) reported an interaction between cytokines and oxidants, where they observed the role of TNF-α, IL-1, and IFN-γ in ROS production. Janssen-Heininger et al. (61) Sidoti-de Fraisse et al. (62), and Loukili et al. (63) reported that ROS and TNF-α have a synergistic effect on cell apoptosis via active transcription factors, nuclear factor kappa-light-chain-enhancer of activated B cells (NF-kB), and activator protein-1 (AP-1). The increased pro-inflammatory expression, NF-kB, PGE_2_, iNOS, and COX-2 are unique in patients with schizophrenia compared to bipolar patients or healthy controls (64). Oxidative stress reduces the levels of antioxidants such as glutathione (GSH) (65–69) and glutamate release from microglia (70). Depleted GSH levels cause NMDA hypofunction in inhibitory GABAergic interneurons (71), which fail to mediate inhibitory and excitatory balance of the microcircuitry, resulting in the loss of synapses or neuronal death (72). Incomplete reduction of oxygen generates superoxide anion (${\text{o}}_{2}^{-}$), which is converted to hydrogen peroxide (H_2_0_2_) by superoxide dismutase (SOD). SOD is an antioxidant enzyme that prevents oxidative damage from hydroxyl radicals and lipid peroxidation (73). A meta-analyses (74) confirmed that there is a decrease in SOD activity in patients There is an interaction between cytokines, oxidants, and microglia, as TNF-α and NADPH oxidase have been observed to activate microglia in patients with schizophrenia (75–79).

**Figure 4:**
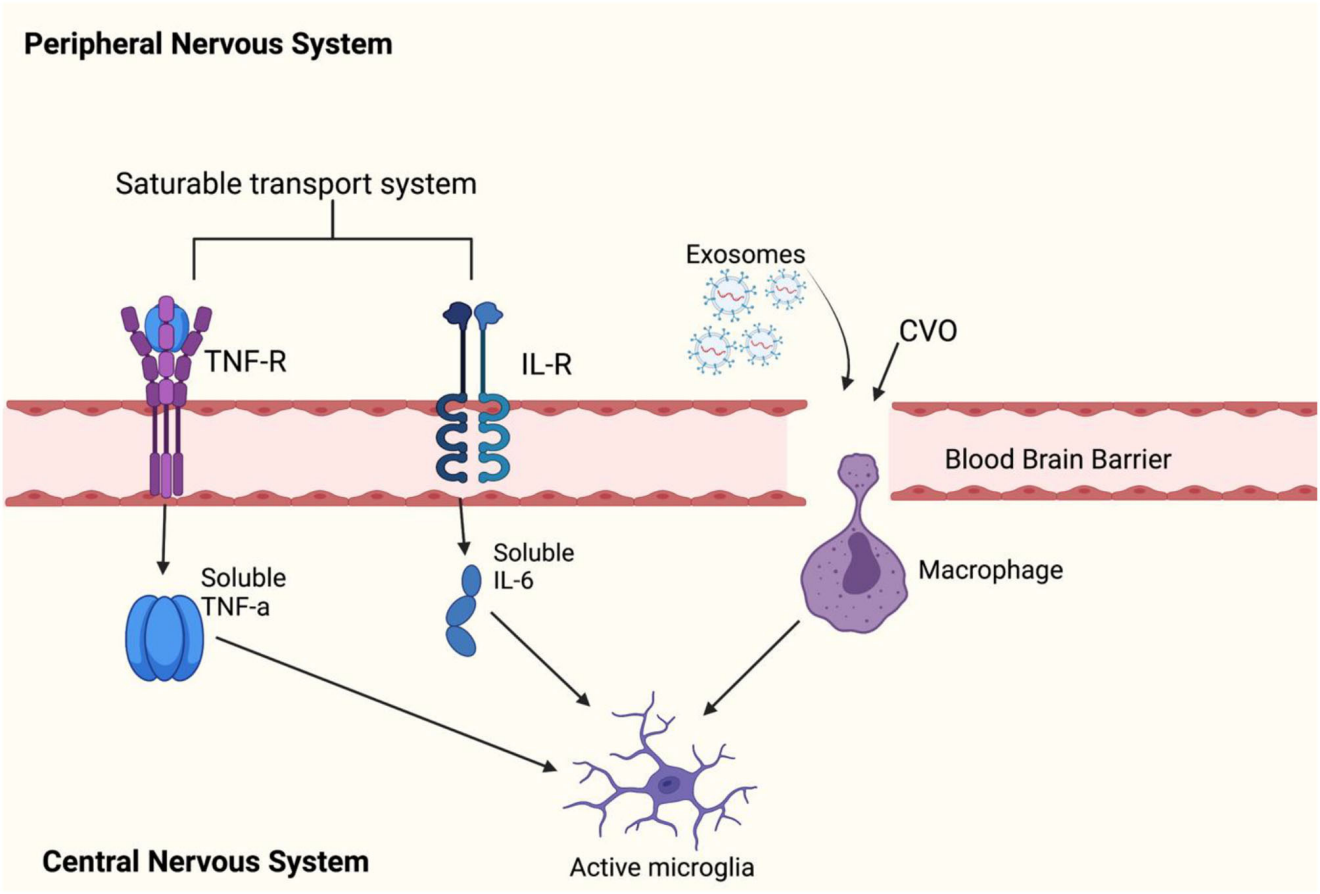
This figure shows how peripheral cytokines can enter the blood brain barrier (BBB) to activate microglia. Peripheral cytokines may activate microglial cells in the brain by passing through the BBB. Previous reports have found that TNF-α, IL-1α, and IL-1B can enter the brain through saturable transport systems (81, 82) or through areas of the brain where the BBB is incomplete such as circumventricular organs (CVOs) (83), and lastly, circulating cytokines can activate IL-1 receptors located on perivascular macrophages and endothelial cells of brain venules (84), which may allow entry of IL-1B cytokines to the brain. There are reports of exosomes easily crossing the BBB (85). However, it is not known whether exosomes directly influence inflammation by activating microglia. Leukocytes can cross the BBB and secrete exosomes, but a (86) has debunked this theory. The (86) study has showed that exosomes can be carried by hematopoietic cells into the blood circulation and released into the brain cells. This can be taken up by membrane receptor-mediator (87) or by phagocytosis (88). However, it is not known whether exosomes directly influence inflammation by activating microglia. Leukocytes can cross the BBB and secrete exosomes; however, a study has debunked this theory.

A meta-analysis observed a neuroinflammatory imbalance in patients in the early stage of schizophrenia (46, 47). There have been alterations in inflammatory markers such as cytokines, reactive oxygen species (ROS), reactive nitrogen species (RNS), and nitrogen oxygen species (NOS) (47). This section provides evidence for altered neuroinflammatory markers in schizophrenia and links it to neuronal and microglial cells. Inflammatory markers play an important role in regulating flush response and microglial activation. Furthermore, it has been observed that the cytokine subtype released (Figure 2) and oxidants levels (Figure 3) regulates the activation of microglia (80). This may raise questions as to how peripheral cytokines may enter the brain. It may be assumed that patients with schizophrenia have poor blood-brain barrier; however, cytokines can enter the brain in different ways (Figure 4). The brain is vulnerable to oxidative stress, such as ROS, NOS, and superoxide species (75). Oxidative stress is defined as the imbalance between pro-and anti-oxidative processes, and there is an imbalance of oxidative stress throughout the different stages of schizophrenia (74, 89–91). Likewise, there is evidence of abnormal antioxidants in the peripheral blood (92–94), CSF (65) and post-mortem brain tissue (74, 95) of patients with schizophrenia. In conclusion, this evidence suggests that the lack of balance between the pro-oxidant and anti-oxidant may contribute to the neuronal abnormalities observed in schizophrenia patients.

### Microglia Activation

The microglia hypothesis (43, 44) suggests that activated microglia are present from prenatal infection to adolescence. When the immune system is challenged, microglial cells are exacerbated, and therefore, prolonged microglial hyperactivity causes cellular or neuronal apoptosis (96). The two-hit process supported by this hypothesis may explain why people who may have exposure to infection in childhood may not go to develop the illness.

Microglia have been shown to be more activated in schizophrenia than in control subjects (97). Studies using positron emission tomography (PET) and peripheral benzodiazepine receptor ligand, (11)C-(R)-PK11195, detected microglial activation in the hippocampus (38) and grey matter (97) of patients with schizophrenia. Bloomfield et al. (98) observed that ultra-high-risk individuals showed increased microglial activation.

## Neurons

**Figure 5:**
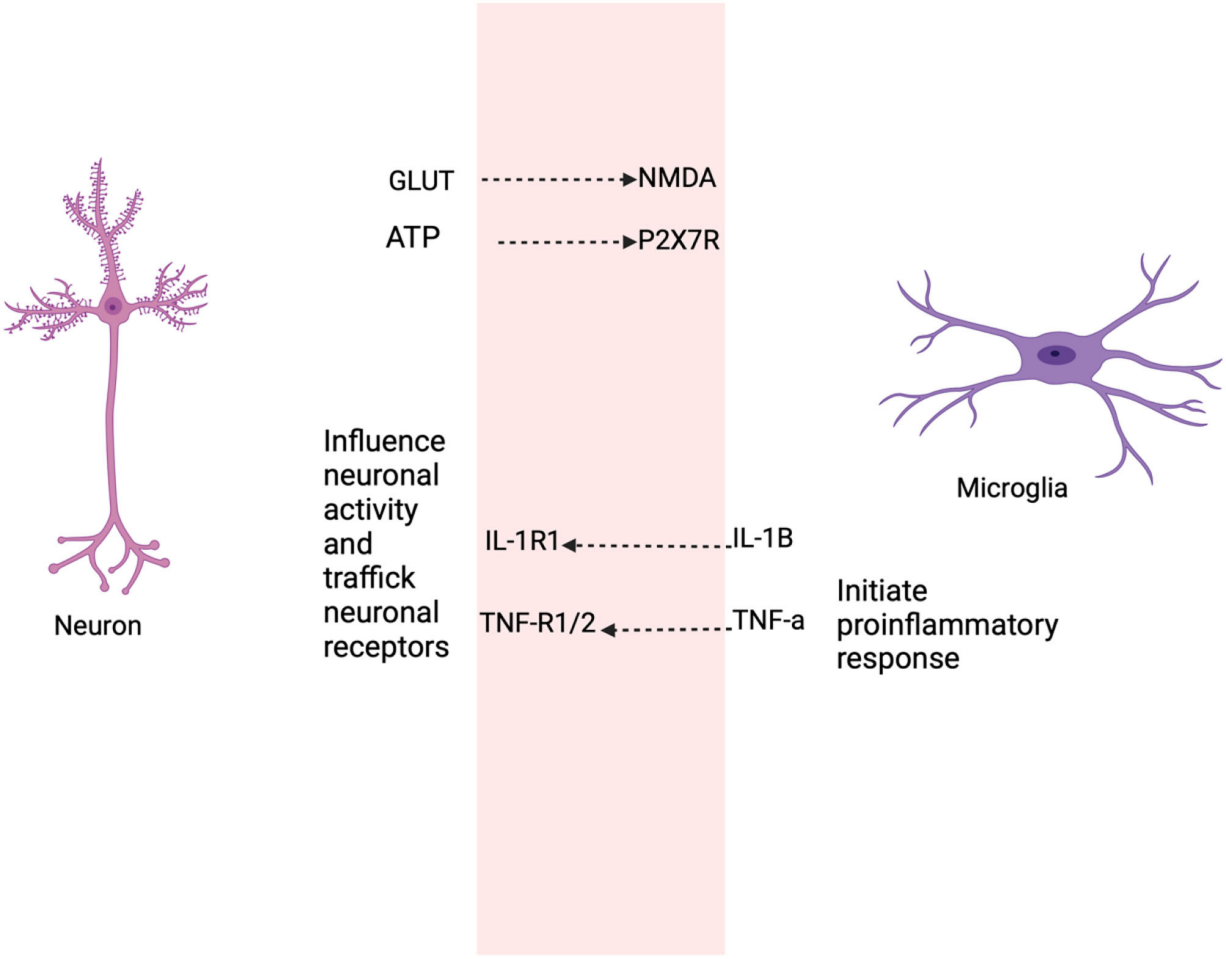
This figure shows the selective roles that neurons and microglia share as a result of the diminished GPR109A-COX pathway. Neurons secrete soluble factors such as cytokines to regulate or maintain microglia activation sites (105–107), the release of neurotransmitter, such as glutamate from neurons influence microglial motility (108, 109). Adenosine triphosphate (ATP) in microglia mediates through P2X7 receptor and produce pro-inflammatory cytokine (110). Likewise, active microglia secrete cytokine, prostaglandin which modulate neuronal function. For example, low levels of IL-1B are required for long term potentiation (LTP) (111, 112), while basal levels of TNF-α are necessary for AMPA and GABA_A_ receptor trafficking (111). IL-1B and TNF induce neurotoxicity through elevated glutamate production resulting in neuronal excitotoxic death (113, 114).

Patients with schizophrenia have a selective loss of grey matter volume, including the left superior temporal gyrus (STG), left Heschl gyrus (HG), left planum temporale (PT), and reduced spine density in the frontal cortex and hippocampus (99–103). The frontal cortex and hippocampus are associated with cognitive functions and reduction in neurons in brain regions, resulting in the cognitive deficits observed in schizophrenia (104). In the cellular pathology of diminished flush response, there are elevated levels of IL-1B and TNF-α, which might mean that microglia are activated. Active microglia and increased pro-inflammatory levels alter the functioning role of LTP, and AMPA and GABA receptors result in neuronal damage. Cognitive deficits may be due to impaired microglia-neuronal function, as microglia and neurons share bidirectional communication (Figure 5).

## JNK

**Figure 6:**
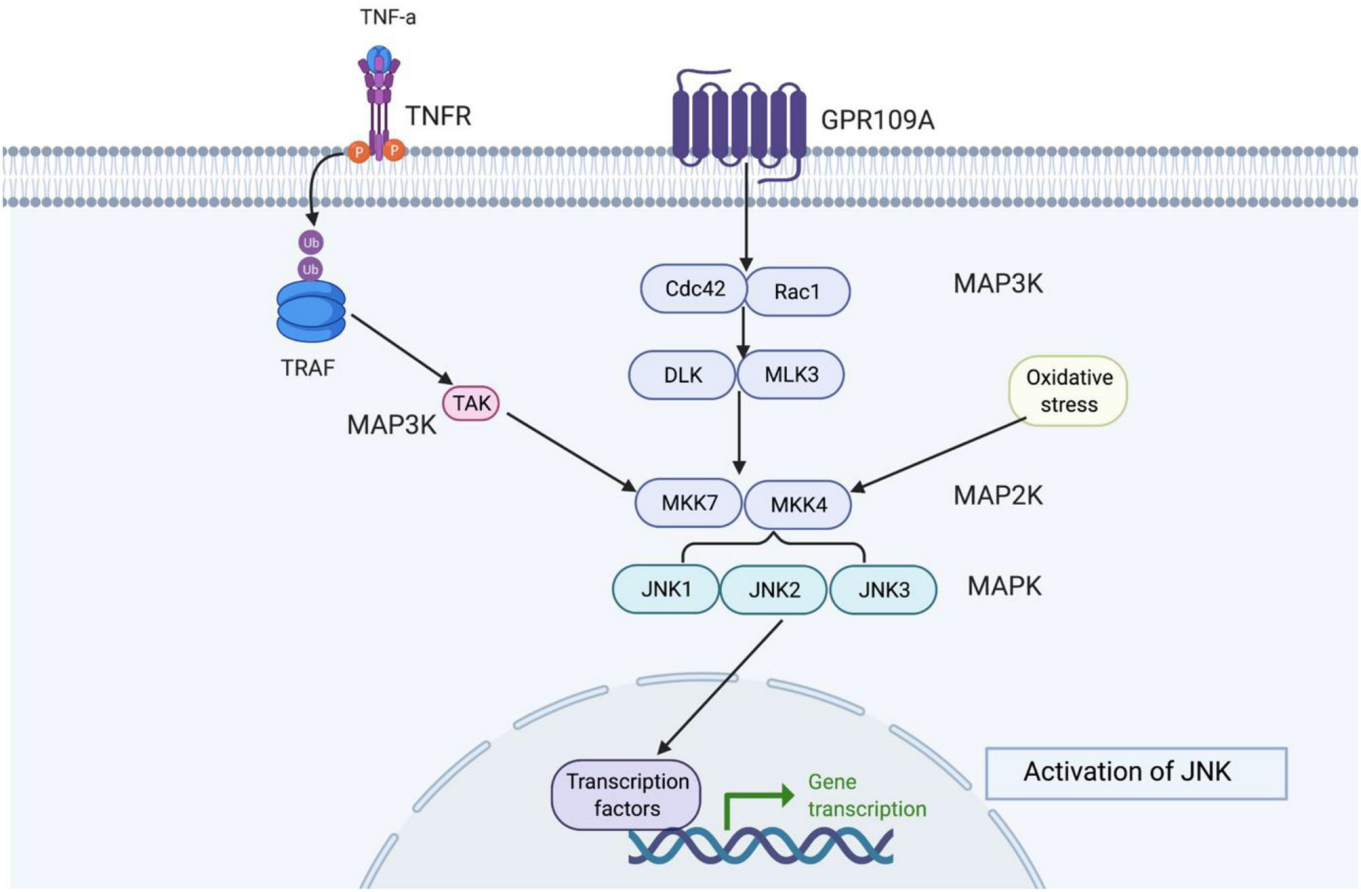
This figure shows how JNK functions in the signalling cascade. Depending on the extracellular signal, there are different MAPKKK proteins, but MAKK and MAPK remain constant. MAPKKK is always a precursor to MAPKK and MAPK, respectively. c-Jun N-terminal kinases (JNKs) belong to stress-activated protein kinases (SAPKs) and are part of the MAPK signalling cascade group, which are involved in signal amplification (118). JNK are activated by stress signals such as hyperosmolarity and heat shock, oxidative stress, UV and ionising radiation, pro-inflammatory cytokines, TNF-α, and IL-1B (119, 120). JNK activity is highest in the brain compared to other non-neural tissues (121, 122). JNK protein are encoded by three genes, JNK1 (Mapk8), JNK2 (Mapk9), and JNK3 (Mapk10). Where JNK1 and JNK2 are expressed ubiquitously in all mammalian tissues and JNK3 is restricted to the heart, testis, and brain (123). The highest level of JNK1-3 mRNA are found in the neocortex, followed by the hippocampus, thalamus, and midbrain (124). Downstream JNK are transcription factors such as c-Jun, c-fos JunD, ATF-2, and ELK-1, which can become activated when exposed to stress signals (125).

**Figure 7:**
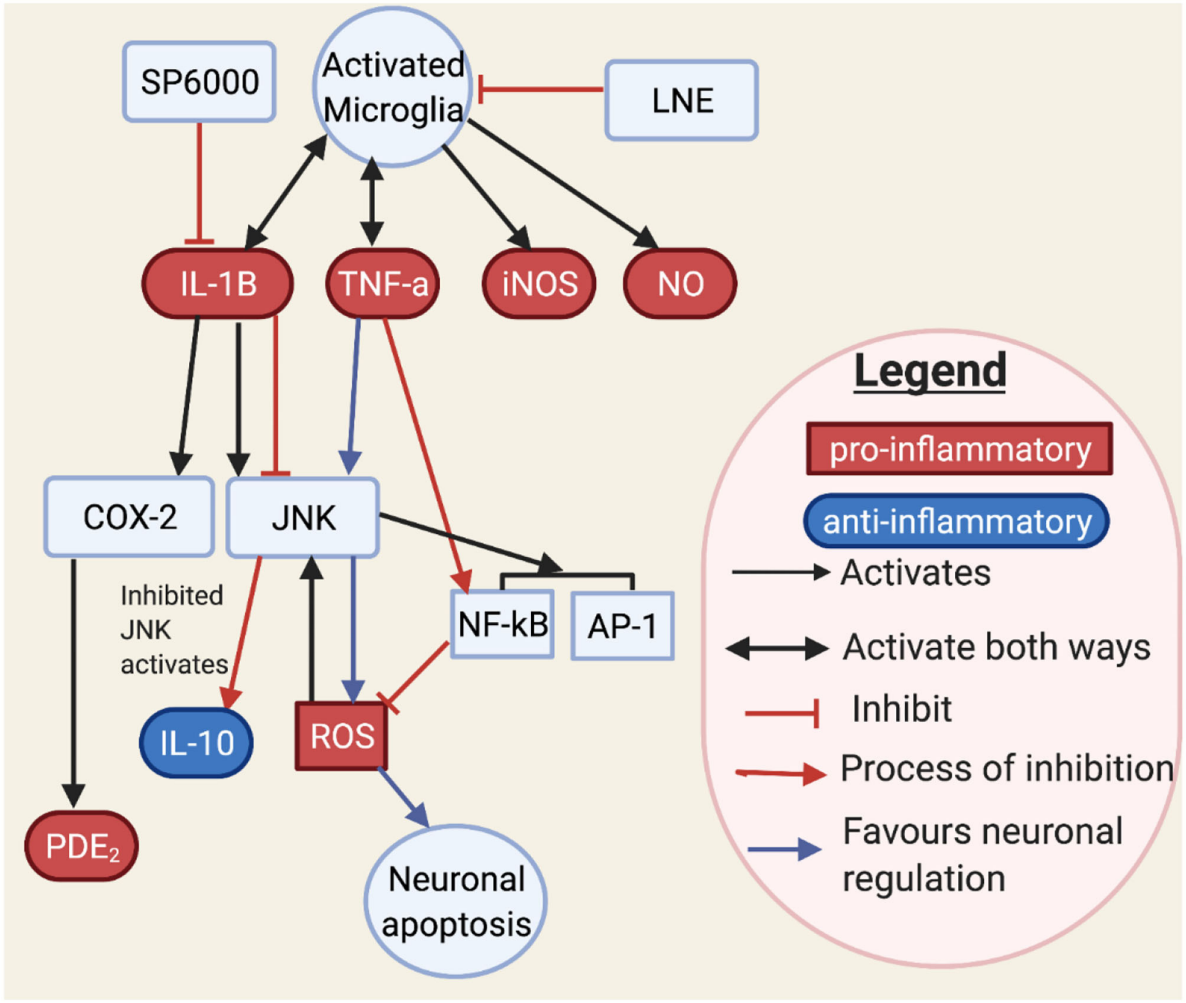
This figure shows how JNK may interact with neurons and microglia through cytokines and transcription factors. JNK controls inflammatory mediators such as IL-1B, TNF-α, iNOS, and NO (126–128). Activated JNK has been involved with cytokine, oxidative species, and transcription factors. TNF-α stimulates JNK, which in turn stimulates ROS. However, ROS may in turn stimulate JNK. It is known that TNF-α stimulating JNK would result in neuronal apoptosis. Moreover, NF-kB when stimulated by TNF would inhibit ROS (127). An aromatic herb, lindera neesiana kurz (LNE), used as an anti-inflammatory substance, reduces pro-inflammatory expression in LPS stimulated microglia cells, such as JNK, p-38, NO, iNOS, COX-2 production and pro-inflammatory cytokine related neuronal injury to JNK phosphorylation in microglia cells (116, 129) and suggested that JNK activation, triggers pro-inflammatory mediators such as TNF-α, IL-6, IL-1β, COX-2, iNOS, NO and PGE_2_, and transcription factors such as AP-1 and NF-κB. SP600125 is a JNK inhibitor which inhibits COX-2 activity through IL-1B. Conversely, IL-1B induces both COX-2 and JNK activation (126). This makes IL-1B a main target for JNK. JNK inhibition has also been observed to increase anti-inflammatory markers (116), which may restore the inflammatory imbalance observed in flush response and prevent microglial activated neuronal death (130).

Schizophrenia is a complex disorder that involves disruption of metabolism, neurotransmission, and cell signalling, and requires the coordination of kinase-mediated signalling events. There has been a signalling imbalance, which may be associated with diminished flush in schizophrenia. MAPKs are a family of serine/threonine protein kinases that are directly modified by ROS/RNS. MAPK can be activated by its upstream MAPKK, MAPKKK, or ROS/RNS (115). The MAPK pathway links inflammation and microglial activation (116). The MAPK family consists of the ERK, JNK, and p38 pathways. JNK has been the most affected kinase in the anterior cingulate circuit (ACC) of patients with chronic schizophrenia (117). This review focuses on JNK, and (Figure 6) shows the characteristic profile of JNK. JNK interacts with both microglia and neurons (Figure 7) through inflammatory mediators.

## Niacin-GPR109A Flush Response

**Figure 8:**
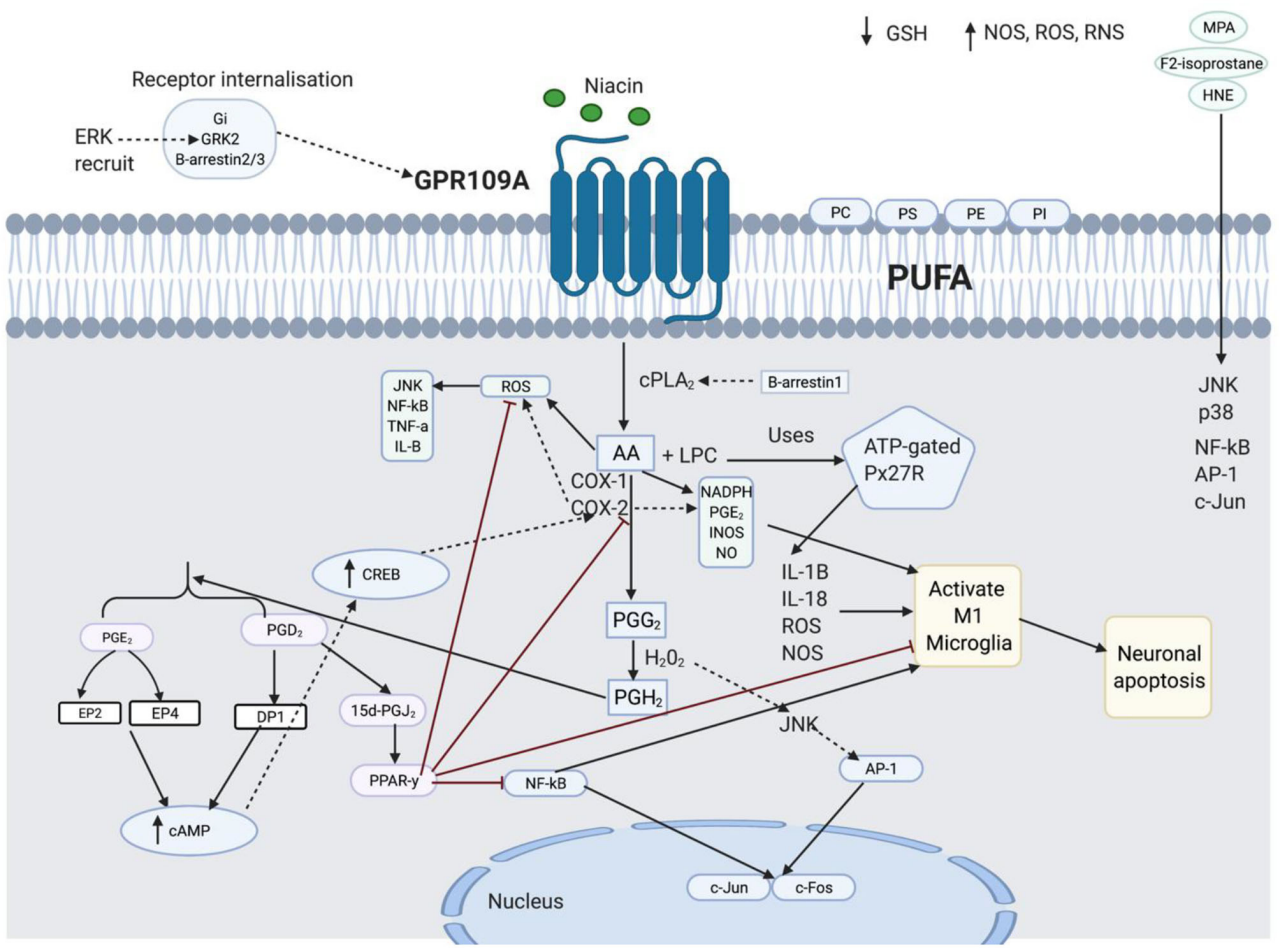
This figure shows the spider-web of inflammation involved in diminished flush response. The details are separated into four subheadings: lipid peroxidation and inflammation, the role of 15d-prostaglandin J_2_ (15d-PGJ_2_) and peroxisome proliferator-activated receptor-y (PPARγ) in anti-inflammation, Transduction Signal role in GPR109A components, and Function of enzymes in diminished flush response and inflammation.

PGD_2_ and PGE_2_ are potent vasodilators, and studies have linked them to diminished flush responses (27, 131). However, it is not fully understood how they are reduced in patients with schizophrenia. In addition, niacin is an antioxidant in many diseases and has a high affinity for its receptor, GPR109A (132–134). It is not well understood why niacin binding to GPR109A is ineffective in lowering the levels of pro-inflammatory mediators observed in schizophrenia. This indicates that there are other potential mediators associated with this aberrant response. This section will discuss (Figure 8) and explore inflammation involvement with the cellular mechanism behind the diminished flush response. It explores the link between the GPR109A-COX-prostaglandin pathway and inflammatory mediators, all of which are relevant to cellular biology behind diminished flush, signal transduction, and inflammation.

### Lipid Peroxidation and Inflammation

Membrane phospholipids contain polysaturated fatty acids (PUFAs), which have a high content of n-6 arachidonic acid (AA) (135). PUFAs contain phosphatidylethanolamine (PE), phosphatidylserine (PS), phosphatidylcholine (PC), and phosphatidylinositol (PI) (136). An inflammatory phospholipid, lysophosphatidylcholine (LPC), is generated when cPLA_2_ cleaves the acyl ester bond of PC (137). LPC uses ATP-gated P2X7 receptor (P2X7R), which is abundant in microglia, to induce IL-1B, IL-18, ROS, and NOS and activate microglia (138). PUFA exposure to oxidative stress is called lipid peroxidation, which induces ROS (139). NOS, ROS, and RNS activate pro-inflammatory mediators, NF-kB, and AP-1 (180) and mediate GSH deficiency (72). The inflammatory imbalance activates NF-κB. Active NF-κB can activate AP-1, which regulates the transcription of Jun and Fos, which are responsible for cell growth and differentiation (140, 141). In contrast, transcription factors may be regulated by ROS-stimulated MAPK (142). This means that the transcription factor may be controlled by MAPK independent of the oxidative species. HNE, a biomarker of lipid peroxidation, has been observed to activate both NF-κB, AP-1, and c-Jun expression, and cell signalling pathways, JNK and p38, when exposed to ROS (143, 144, 180). Therefore, pro-oxidants activate HNE-induced activation of the cell signalling pathway.

### (15d-PGJ_2_) and (PPARγ) in Anti-inflammation

15d-PGJ_2_ increased the transcriptional activity of PPARγ. This downregulates the pro-inflammatory markers, COX-2, iNOS, AP-1, Stat-1, NF-kB, TNF-α, IL-1β, and PGE_2_, and increases antioxidant enzymes, hemeoxygenase-1 (HO-1) and GSH by PPARγ and 15d-PGJ_2_, respectively. PPAR-*y* and 15d-PGJ_2_ negatively regulate microglial activation and prevent neuronal apoptosis (145–153). NF-κB may be activated by ROS, cytokines, JNK (154), AP-1, and COX-2 (155). 15d-PGJ2 participates in the feedback mechanism (156) by PPAR*y*, which inhibits activated NF-κB by increasing IKB expression (157, 158). PPAR-*y* activates antioxidant enzymes such as SOD, HO-1, and GSH to reduce ROS.

### Transduction Signal Role in GPR109A Components

Gi, GRK2, and B-arrestin 3 are important for receptor internalisation (159). Upon activation of the GPR109A receptor by niacin, the G_i_ subunit is released from the GB*Y* subunit, followed by desensitisation, which catalyses and phosphorylates the activated receptor by G protein-coupled receptor kinase (GRK2). Activated GPR109A promotes translocation and binding of B-arrestin 3 to the plasma membrane, resulting in receptor internalisation (159). Gi is involved in GRK2 recruitment to phosphorylate the C-terminus of GPR109A and subsequent ERK1/2 activation (160, 161). Phosphorylated ERK1/2 has been observed to potentiate GRK2 activity, resulting in the inhibition of leukocyte migration. In comparison, p38 blocks GRK2 function and facilitates cell migration (162, 163). The ERK pathway uses GRK2 to activate GPR109A; conversely, the ERK pathway is GPR109A independent, when activating B-arrestin 1. B-arrestin 2 phosphorylates and activates JNK3 in endosomes (164). It has also been observed that disrupted, ubiquitinated B-arrestin 2 promotes NF-κB signalling (165). ERK has been associated with B-Arestin 1, whereas it may be inferred that B-arrestin 2 may be associated with the JNK pathway, as it is both a precursor for c-Jun and an activator of NF-kB.

### Function of Enzymes in Diminished Flush Response and Inflammation

Phosphorylated cPLA_2_a releases AA to induce pro-inflammatory markers, NADPH oxidase, superoxide, PGE_2_, INOS expression, and NO production, which activate microglia cells (166, 167). AA release produces ROS as a by-product, which activates JNK, NF-κB, TNF-α, and IL-1 to further activate COX-2 (168–171). Overactive COX-2 increases pro-inflammatory, iNOS, PGE_2_, nitric oxide, and peroxynitrite anions, which attack membrane phospholipids and lower their antioxidant defence (172). There was a synergistic effect between COX-2 and PGE_2_ expression; an increase in one would increase the expression of the other. PGE_2_ acts as a pro-inflammatory mediator and increases M1 microglial activation by increasing COX-2, IL-1B, and IL-6 levels (173–176). Active PGE_2_ activates the EP_2_ receptor, which increases cAMP production and activates cAMP response element-binding protein (CREB), which is responsible for increasing COX-2 expression (177–179). Different receptors induce different functions; for example, EP_2_ receptors regulate TNF-α, whereas EP_4_ receptors mediate IL-1B secretion (173). JNK inhibitor is known to reduce COX-2 expression, mediated by IL-1B, and it may be questioned whether this is also mediated through the EP_4_ receptor. H_2_0_2_ partially activates JNK (180) and AP-1 protein (181) to increase c-Jun and c-Fos (182) and resulting in cell apoptosis.

## Phospholipid Abnormality

The membrane phospholipid hypothesis suggests that the abnormality observed in schizophrenia may be due to altered phospholipid metabolism (183, 184). LPC levels are disrupted in schizophrenia (185–187). LPC inflammatory activity is controlled by NLRP3 and NLRC4 genes (110).

## Fatty Acid Abnormality

Fusar-Poli and Berger (188) showed reduced PUFA levels in patients with schizophrenia. PUFA is responsible for both membrane fluidity and its ligand-receptor interaction; it increases the concentration of receptors in the membrane and allows the ligand to interact with the receptor (189). Disrupted ligand-receptor interaction might be a reason for the reduced binding between GPR109A and its ligand, niacin, and therefore, its inability to release PGD_2_ and PGE_2_, resulting in a diminished flush response. Niacin has anti-inflammatory properties, and less exposure to niacin may contribute to the inflammatory imbalance observed in schizophrenia. Smesny et al. (190) suggested that structural changes observed in grey matter may be due to lipid membrane alterations, and that antipsychotics may influence lipid metabolism. A meta-analysis (191) showed that PUFA supplement intake and omega-3 or 6 reduced TNF-α levels and delayed onset of illness in ultra-high-risk patients with schizophrenia (192).

### Biomarkers of Lipid Peroxidation

Lipid peroxidation is described as an oxidant that attacks PUFAs by inserting oxygen into the carbon-carbon double bond and altering the membrane structure (193). Lipid peroxidation can form secondary products such as malondialdehyde (MDA), propanal, and 4-hydroxynonenal (4-HNE) (91, 194). It has been observed that 4-HNE at low levels is metabolised, and therefore maintains a homeostatic environment, but at high levels, it can cause cell death and damage cell signalling proteins (195). HNE increases intracellular calcium levels in neurons (196), which may activate MAPK proteins, activate the COX pathway, or induce neuronal toxicity (197). Uchida et al. (180) confirmed that JNK is an important signalling mediator in cellular defence against toxic products generated from lipid peroxidation. MDA is a specific biomarker for lipid peroxidation in omega-6 fatty acids (198). MDA exposure alters membrane fluidity, resulting in the loss of membrane integrity (199). However, there is a heterogeneous distribution of MDA in schizophrenia, which may be due to confounders such as antipsychotics, which were not separated in the study (200). The sensitivity of biomarkers can also be an issue when measuring lipid peroxidation. There have been reports of increased F2-isoprostane (201) and microRNAs (miRNAs) in schizophrenia (202–205), which are more sensitive biomarkers of lipid peroxidation (201, 206–208).

### Arachidonic Acid

Glen et al. (209), McNamara et al. (210), and Yao et al. (211) reported AA depletion in red blood cells (RBCs) in patients with schizophrenia. There is a controversy about the cause of the depleted AA; some researchers suggest that it may be due to niacin blunted response (212), whereas others would argue that niacin blunted response has been observed at normal AA levels, and instead may be due to disrupted AA metabolism (213). Skosnik and Yao (11), Horrobin (214), and du Bois et al. (215) suggested that oxidative stress reduces AA levels and modifies the signal transduction pathways to cause neuronal damage, as observed in schizophrenia. Cao et al. (216) and Covault et al. (217) reported that increased long-chain fatty acid-CoA ligase, type 4 (FACL4) activity as a result of genetic mutation leads to more rapid sequestration of free AA, resulting in reduced AA.

### AA and JNK

In phagocytic cells, AA translocates activated rac from the cytosol to the membrane to activate NADPH oxidase and activate JNK, respectively (218–220). However, it has been observed that JNK activation is independent of AA metabolism. Minden et al. (221) showed that the antioxidant N-acetylcysteine blocked two-thirds of AA-induced JNK activation. It may be inferred that activated JNK is more dependent on oxidative species than AA.

## Prostaglandin

A systemic imbalance of pro-inflammatory and anti-inflammatory prostaglandin levels has been reported in patients with schizophrenia (222). This imbalance may be associated with altered mediators involved in the niacin-GPR109A-COX pathway. The degradation of phospholipid membranes into eicosanoids results in the production of free radicals, which may contribute to the imbalance (223).

### PGD_2_ and PGE_2_

Morrow et al. (224) used gas chromatography-mass spectrometry to detect large levels of PGD_2_ and its metabolite 9a,11 β-PGF_2_ following oral niacin. However, (225, 226) suggested that flushing is strictly related to PGE_2_. Furthermore, (227) suggested that increased cAMP production by their receptors, DP_1_, EP_2_, and EP_4_, contributes to flushing. However, Wise et al. (228) countered earlier studies by showing that DP_1_ and EP_2_ receptor knockout showed 40 and 20% reduced flushing, respectively. In addition, laropiprant, which is an antagonist with high selectivity for DP_1_, showed reduced flushing when compared to placebo, but 70% of the time, the participants still had flushes (229). This suggests that PGD_2_, PGE_2_, and their receptors are important in the flushing response, but partially contribute to its effect.

Moreover, PGE_2_ is synergistic with COX-2 to activate microglia (173–176), and active microglia can damage neurons. COX-2 inhibitors serve as neuroprotectants by reducing PGE_2_ levels (230). High concentrations of PGD_2_ have also been observed to be neurotoxic (231, 232). This is interesting because the diminished flush effect resulted in low PGD_2_ levels. PGD_2_ exerts anti-inflammatory properties through PPAR-*Y*; therefore, it may be suggested that high PGD_2_ would be beneficial for cells. Furthermore, Liang et al. (233) cleared our understanding by stating PGD_2_ concentration of 1 nM-1 μM, and PGE_2_ at concentrations of 0.01–1 μM are neuroprotective.

### PGE_2_ Level Controversy

Cytosolic PGE_2_ levels were observed to be reduced in the temporal cortex of patients (234). Other studies have suggested that PGE_2_ levels (64, 235–238). Pierre et al. (239) and Quraishi et al. (240) showed that PDE_2_ can be modulated by peroxisome proliferator-activated receptor γ (PPAR*y*), a nuclear receptor stimulated by prostaglandin J_2_ (PGJ_2_). As PGE_2_ is a pro-inflammatory mediator, this may suggest that PPAR*y* may regulate both pro- and anti-inflammatory properties based on its interaction with the prostaglandin type. A recent study, which considered the acute phase of schizophrenia, eliminated potential confounders such as drug dependency, alcohol consumption, development delay, and dementia, and matched patients based on their age, sex, marital status, education, and onset of illness, confirmed that there are lower serum levels of PGE_2_, 15d-PGJ_2_, and PPAR*y* levels in patients (241). In contrast, Martínez-Gras et al. (222) showed reduced levels of 15d-PGJ_2_, PPAR*y*, and IkBa, but increased levels of PGE_2_. However, participants in the study had been using antipsychotic drugs and did not match the severity of the illness. The variation in PGE_2_ levels may depend on the severity of illness and the use of antipsychotic drugs.

### PPAR*y* and 15d-PGJ_2_ Role

PGD_2_ can be degraded non-enzymatically to form a J-series, 15-deoxy-Δ^12, 14^-PGJ_2_ (15d-PGJ_2_), which binds to PPAR-γ (242, 243). 15d-PGJ_2_ is a cyclopentenone prostaglandin, which reportedly exerts anti-inflammatory effects on microglia (150). 15d-PGJ_2_ is the first endogenous ligand of PPAR-γ. PPAR-γ plays an important role in lipid metabolism, inflammation, proliferation, and differentiation of cells. Furthermore, PPAR*y* is considered a negative regulator of activated macrophages, and can stimulate or inhibit 15d-PGJ2 gene expression by altering transcription factors, AP-1, STAT, and NF-kB (148, 158). To reverse macrophage activation, transcription factors are downregulated by PPARγ. PPAR*y* regulates the relationship between microglia and neurons by modulating cytokines IL-18 expression in microglia, which has an inhibitory effect on LTP. PPAR*y* agonist reverses IL-18 mediated attenuation of LTP by enhancing synaptic plasticity (148, 244). JNK inhibitors are also known to act as PPAR*y* agonists, supporting their anti-inflammatory role (245–248).

## G-Coupled Receptor

PUMA-G in mice is an orthologue of the human GPR109A receptor. Mice lacking PUMA-G did not release PGD_2_ or PGE_2_, and therefore, did not show flushing (35). The alteration of receptor components has been associated with diminished flush. B-arrestin is used for cell signalling, receptor desensitisation, and internalisation (249). Internalisation is involved in receptor desensitisation and signalling, and contributes to the diversity of GPCR-dependent signalling (250). B-arrestin1 is a biassed agonist because it may induce a flushing response independent of the GPR109A receptor by increasing cPLA_2_ phosphorylation, while depletion of B-arrestin1 reduces activated cPLA_2_ (249). B-arrestin2/3 was significantly reduced in the schizophrenia group compared to that in the control group. Furthermore, reduced GRK in the frontal cortex was observed in both younger and older patients with schizophrenia. However, Bychkov et al. (251) observed a difference in GRK levels in both young and older patients with schizophrenia compared to controls. In young patients with schizophrenia, GRK3 had been reduced, whereas in the older schizophrenia group, GRK6 showed the greatest reduction. It may be inferred that disrupted B-arrestin or GRKs may result in diminished flush response, and confirmed that age is an important factor in schizophrenia.

## Enzymes

**Figure 9:**
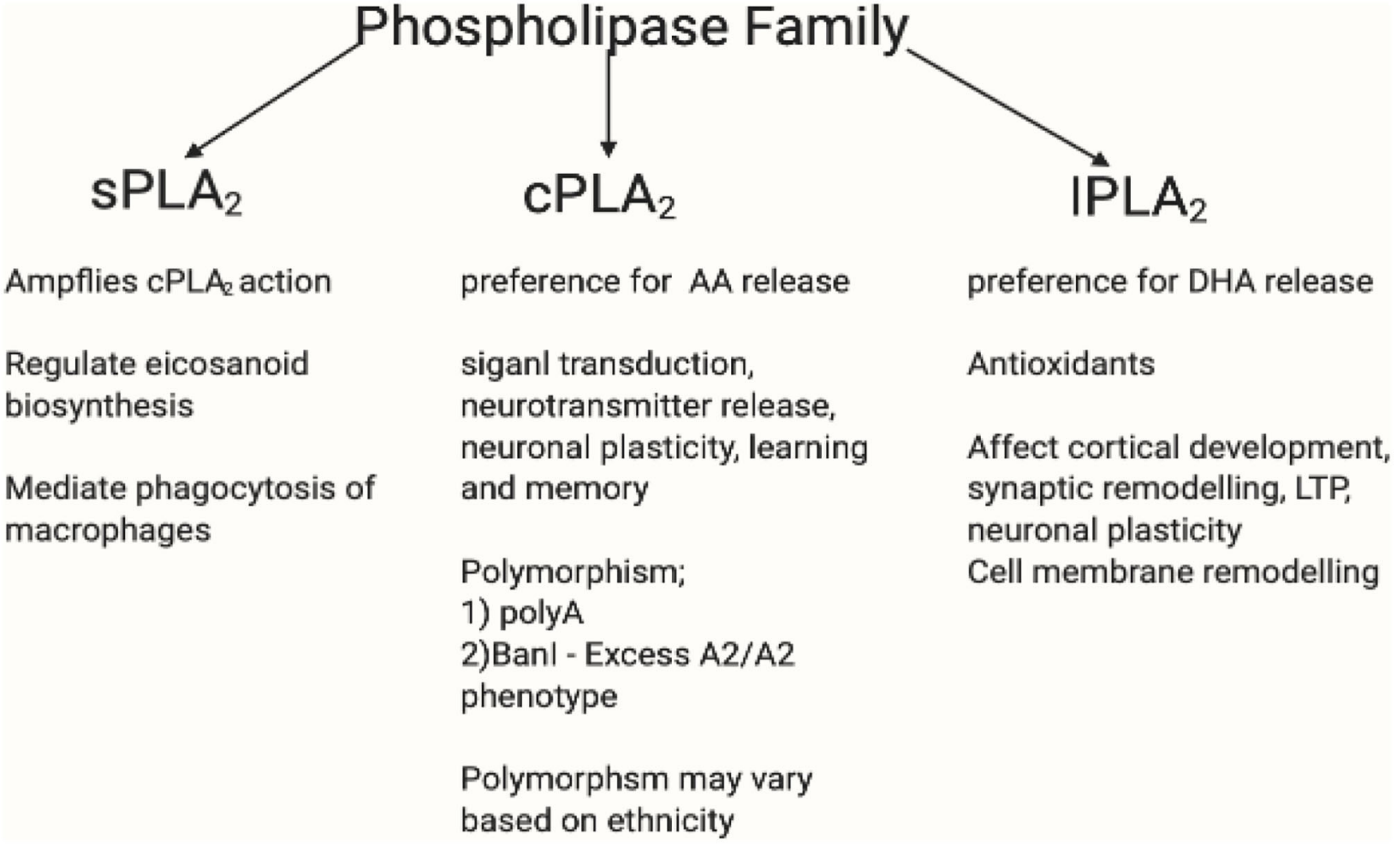
This figure shows the PLA_2_ family profile. The phospholipase A_2_ superfamily consists of enzymes that catalyse the hydrolysis of (sn-2) ester bond of phospholipid to liberate free fatty acids such as AA (254). The family of PLA_2_ consists of secretory calcium dependent PLA_2_ (sPLA_2_), intracellular calcium independent PLA_2_ (iPLA_2_), and the intracellular calcium dependent PLA_2_ (cPLA_2_) (255). iPLA_2_ possesses antioxidant and anti-inflammatory properties and have preference for DHA release. Likewise, cPLA_2_ has preference for AA release (256, 257). iPLA_2_ affects cortical development, synaptic remodelling, long-term potentiation (LTP), neuronal plasticity, and cell membrane remodelling (258). Whereas, cPLA_2_ participates in signal transaction, neurotransmitter release, neuronal plasticity, and learning and memory (259–261). Overexpressed iPLA_2_ did not induce COX-2-dependent PGE_2_ release, but instead mediated PGE_2_ release by COX-1 (262–264). sPLA_2_ amplifies cPLA_2_ action by regulating eicosanoid biosynthesis and mediate phagocytosis of macrophages (265). cPLA_2_ mutation varies in different ethnicities. There is an existing association between niacin flush response and PLA2G4A and PTGS2 gene polymorphism. The PLA2G4A gene encodes a calcium dependent form of cPLA_2_ (266, 267), whereas the PLA2G4C encodes a calcium independent form (268). There had been two polymorphisms of PLA2G4A: polyA and BanI polymorphism occur near the first intron and promoter region, respectively (269). Association between PLA2G4A polymorphisms and disease have been reported (270–272). The difference in BanI alleles between A1 (cut) and A2 (uncut) showed that cPLA_2_ activity with A2A2 genotype was higher than that with A1A2 and A1A1 (273). Excess A2/A2 homozygote has been associated with BanI polymorphism in schizophrenia (272, 274). A Korean study replicated those in western countries which supported cPLA_2_ gene Ban I polymorphism in schizophrenia (275). In a Brazilian population, higher cPLA_2_ activity correlated significantly with G allele of BanI polymorphic site and was associated with a higher risk of developing schizophrenia (273). However, some studies contradict this by reporting the lack of association between cPLA_2_ gene and schizophrenia (276–278). It may be inferred here that while there is disruptive cPLA_2_ gene for schizophrenia in different ethnicity, its polymorphism mutation may vary. Created in BioRender.com.

**Figure 10:**
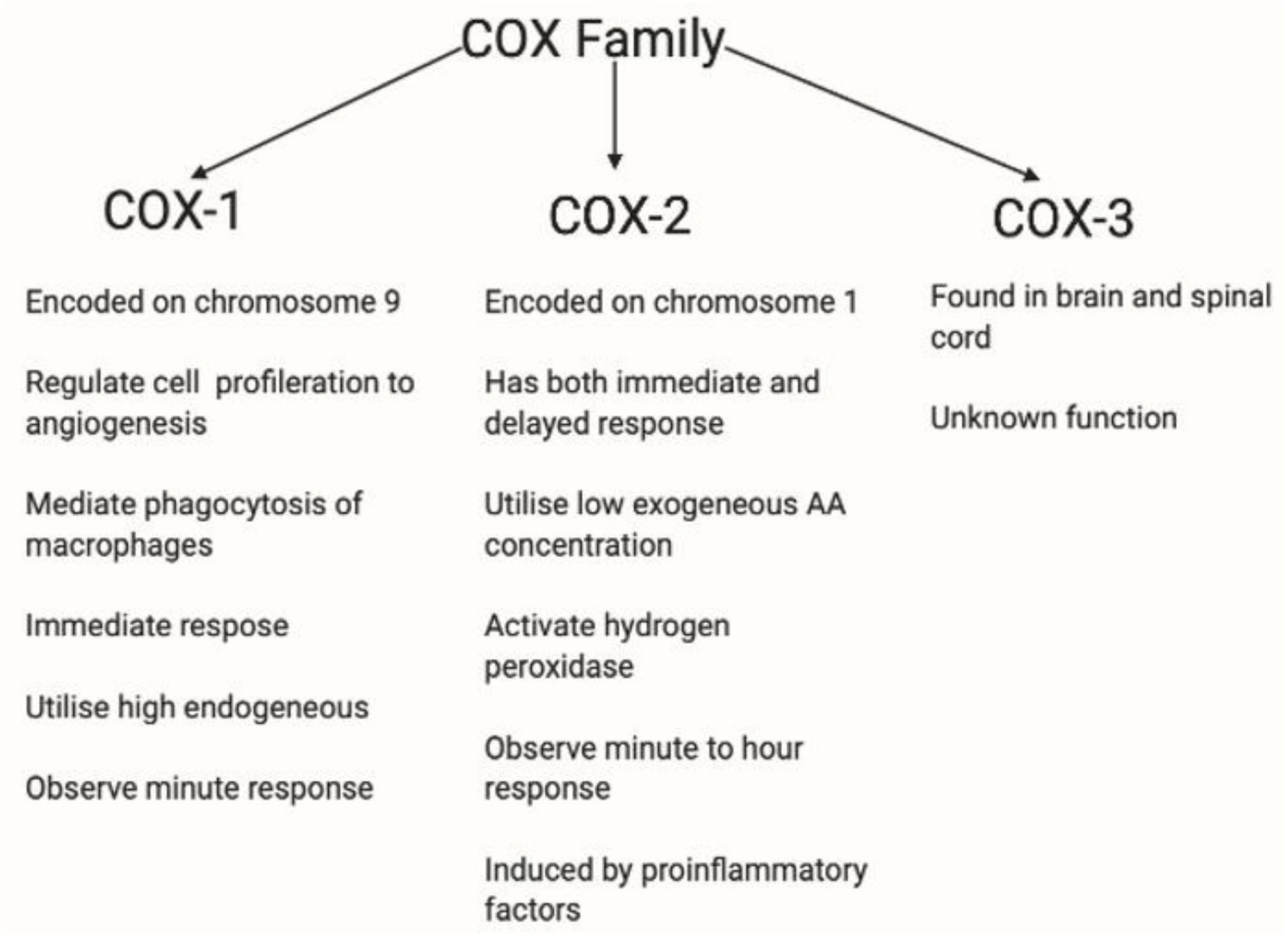
This figure shows the COX profile. There are three isoforms of COX. COX-1 plays a role in homeostasis (279). COX-2 is regulated by growth factors and cytokines such as IL1B, IL6, or TNF-α (280) and is overexpressed in inflammation, and therefore is relevant in this thesis. COX-3 has been used in the brain and spinal cord. However, the functions of COX-3 are currently unknown (281). COX-1 and COX-2 are rate-limiting enzymes in AA-derived prostaglandin production, COX-1 is expressed in most tissues whereas COX-2 is induced by pathophysiological responses by inflammatory stimuli such as IL-1, IL-1B, iNOS and growth factor, and EGF (36, 175, 282, 283). COX-1 is restricted to immediate response, whereas COX-2 is active in both immediate and delayed response. Also, low exogeneous AA concentration been observed and are utilised by COX-2 (284), whereas high endogenous AA concentration are utilised by COX-1 (285). COX-2 has a lower threshold for hydrogen peroxidase activation at low AA concentration (286, 287). Depleted AA is observed in diminished flushing response, and hydrogen peroxidase is relevant in stimulating microglia cells, thus, making COX-2 a relevant enzyme in diminished flush.

Enzymes are biological catalysts that convert essential fatty acids to prostaglandins in the GPR109A-COX-prostaglandin pathway. Horrobin (252) suggested that one of the factors behind diminished flush was dysfunctional enzyme activity, which contributes to reduced prostaglandin levels. Furthermore, the GPR109A flushing response can be ablated by inhibiting PLA_2_ and COX-1/COX-2 activity (253). Figures 9, 10 shows the profiles for the PLA_2_ and COX families, respectively.

## PLA_2_

### Controversy in Phospholipase Activity

There is increased PLA_2_ activity in the cortex and thalamus in patients with first-onset schizophrenia (190). This study provides an insight that PLA_2_ activity is dependent on the stage of illness, and its activity may vary based on the brain region and use of medication. However, the study has limitations, as it did not indicate which PLA_2_ activity is being measured, as different PLA_2_ have different functions and activities.

Dr. Horrobin's membrane hypothesis suggests that elevated levels of calcium-dependent cytosolic group IVA PLA_2_a (cPLA_2_a) observed in schizophrenia are responsible for the depletion of AA (183). Messamore et al. (288) and Kim et al. (289) reported an increase in intracellular calcium concentration, which may result in increased cPLA_2_ activity. Instead, (290–293) suggested that there is increased iPLA_2_ and decreased cPLA_2_ activity in patients with schizophrenia. The iPLA_2_ may be increased as negative feedback by producing an antioxidant that mediates increased oxidative stress, as observed in schizophrenia patients (294–296). The reduced cPLA_2_ has a higher preference for cleaving AA, which may explain the reduced AA levels in patients with schizophrenia. It may be concluded that the variation in cPLA_2_ activity may be due to confounders such as age, medication, disease stage, ethnicity, and other medical status which induce pro-inflammatory and anti-inflammatory imbalances.

### cPLA2, JNK and Its Effect on Cells

Phosphorylation of Ser^505^, Ser^515^, and Ser^727^ activates cPLA_2_ (297, 298). Activated cPLA_2_ cleaves AA and induces the production of inflammatory mediators such as eicosanoids (299–301). There are insufficient studies regarding Ser^515^ and Ser^727^ and their effect on cPLA_2_. However, phosphorylation of ser^505^ on cPLA_2_a increases phospholipase binding to membrane phospholipids at low calcium concentrations, altering the PLA_2_ conformation to ensure a better fit to the catalytic domain of membrane phospholipids (298). Some studies have suggested a relationship between MAPK and cPLA_2_ activity. However, in macrophages, there have been inconsistent reports of ERK1/2 and p38 links in phosphorylating cPLA_2_ at ser^505^ (302–304). Casas et al. (305) used MAPK inhibitors for ERK, p38, and JNK and found that only the JNK inhibitor effectively blocked cPLA_2_a phosphorylation in macrophages. This advances our understanding of the prominent role of JNK in cPLA_2_a phosphorylation in macrophages. Microglia are resident macrophages of the brain, which share similar functional and morphological properties to macrophages (306), therefore it may be inferred that microglia would have similar effects. However, there is no study linking MAPK and cPLA_2_a to neurons, although from our understanding of how microglia and neurons influence each other, there is a possibility that alteration in cPLA_2_ activity in microglia might affect neuronal functions. Furthermore, it has been observed that cPLA_2_ and dopamine are inversely related (307), where increased dopamine levels reduced cPLA_2_. The mechanism is not understood properly, but studies have shown that dopamine and glutamate alternation have specifically affected cPLA_2_ mediated AA release, but not mediators downstream of AA (289, 308).

## COX

### Activators of COX

PGD_2_, PDE_2_ mediated by COX-1 and COX-2, play an important role in the flushing response (35, 228). COX-2 knockout reduces both pro-inflammatory, PGE_2_, and NF-kB (309–311). Deng et al. (312) suggests that overexpression of COX-2 activity has been associated with increased histone acetyl transferase (HAT) and p300 gene, which is located near the NF-kB promoter, deletion or suppression of these transcriptional activators, and reduced COX-2 expression. Future studies need to investigate the link between HAT, p300, and COX-2 overexpression in schizophrenia. Ultimately, IL-1B is a potent inducer of COX and induces the synthesis and activity of PLA_2_ in cells (313). Therefore, it may be used as a target to control both the activation levels of COX and PLA_2_ by JNK.

### COX in Microglia

COX-2 is important for producing inflammatory responses, which can activate microglia (314). During prostaglandin production via the COX pathway, ROS are generated as a by-product, along with the production of inflammatory agents such as cytokines and oxidative stress (282), all of which contribute to microglial activation.

### Inhibitors of COX-2 Expression

When there is a high inflammation level, antipsychotics are less effective in reducing psychosis (315, 316). COX-2 overexpression has been linked to cognitive deficits in schizophrenia; COX-2 inhibition has been shown to have therapeutic effects, particularly when administered in the early stage of the disease (317–324). Mattson et al. (325) Weggen et al. (326), and Morihara et al. (327) suggested that nonsteroidal anti-inflammatory drugs (NSAIDs) regulate NF-κB and can serve as a therapeutic target for several psychiatric disorders. Nitta et al. (319) observed that NSAID celecoxib and risperidone are more beneficial in patients than the administration of antipsychotic risperidone alone. Niederberger et al. (328) and Tegeder et al. (329) showed that patients who used both NSAIDs and antipsychotic drugs had a higher psychotic relapse rate. These reports suggest that NSAIDs may play a controversial role in upregulating COX-2 expression, instead of downregulating COX-2. Harris et al. (330) theory on COX-2 as a double agent may influence the role of NSAIDs or COX-2 inhibitors. COX-2 can also participate in both pro-inflammatory and anti-inflammatory effects. During the development of inflammation, pro-inflammatory (*via* PGE_2_), but anti-inflammatory (via PGD_2_ and 15d-PGJ_2_) during resolution. Therefore, there is a chance that COX-2 inhibitors may instead inhibit anti-inflammatory properties, therefore, exacerbating schizophrenic symptoms. Therefore, alternative methods should be explored to ensure the selective downregulation of overactive COX-2 expression.

Increased COX-2 expression is dependent on MAPK activation (331). Yang et al. (332) showed that IL-1B induction is responsible for elevated COX-2 expression in hippocampal neurons. Rösch et al. (331) showed fibroblasts released PGE_2_ when stimulated with IL-1B, were also found to have overexpressed COX-2 and defective JNK signalling. To confirm this finding, the JNK inhibitor, SP600125, along with IL-1B, lowered both PGE_2_ and COX-2 expression (333–336). It may be inferred that schizophrenia patients with overexpressed COX-2 may present with increased levels of pro-inflammatory mediators. Therefore, to maintain inflammatory balance, the JNK inhibitor SP600125 may be administered, which may downregulate pro-inflammatory mediators. Other inhibitors such as glucocorticoids and minocycline have been shown to downregulate AP-1 or NF-κB in microglial cells and protect against neurotoxicity, while improving cognitive and negative symptoms of schizophrenia (337, 338).

### Hydroperoxide

Stimulated hydrogen peroxide produces NADPH oxidase, otherwise known as phagocytic oxidase (PHOX), which converts microglia to an activated or cytotoxic state (339).

## Exosomes

Exosomes transmit genetic information between cells, and miRNAs are found inside exosomes. These exosomes can be secreted by neurons or astrocytes (340). These exosomes circulate around the body to nearby and distant cells (341). Exosomal miRNAs have also been shown to be involved in the inflammatory response (342). A recent study found an association between dysregulated exosomes and schizophrenia (343). Du et al. reported a pattern between dysregulated exosomes and glycerophospholipid metabolism. The relationship between exosomes and GPR109A receptor should be investigated in future studies.

## Genes

Schizophrenia is caused by the cumulative effects of risk variants in over 100 genes (45, 344). Most these genes are associated with neurons, neurotransmitters or synaptic plasticity (345–347). Table 1 attempts to match the current GWAS for schizophrenia with genes which may be involved in the diminished flush response. A negative result may be a false negative, whereas a positive match may be false positive. As observed in the table, GPR109A has been a match, which may suggest that risk variants in GPR109A may contribute to the aetiology of schizophrenia, as well as to an abnormal flushing response. GPR109A showed a positive match, indicating genetic mutation. This matched with the review analysis which suggested that there is an alteration in the receptor protein conformation and components, B-Arestin and GRK. We would have expected alternation in cPLA_2_ and COX-2, as there had been strong evidence in this review suggesting alterations in its genetic, protein expression, and activity. The dual role of PPARG in inflammation and its reduced expression in patients with schizophrenia would make it a good target. We would not expect much alteration in prostaglandin enzymes and receptors, as strong evidence suggests that they do not significantly contribute to the flushing response.

**Table 1:** Genes in GPR109A-COX-prostaglandin pathways matched against 128 GWAS schizophrenia.

**Gene**	**Aliases for gene**	**(GRCh37/hg19)**	**128 GWAS Chr. position**
HCAR2	Hydroxycarboxylic Acid Receptor 2 (GPR109A)	chr12:123,185,840–123,187,904	Yes
PLA2G4A	Phospholipase A2 Group IVA (cPLA_2_)	chr1:186,798,032–186,958,113	No
PLA2G6	Phospholipase A2, Group VI (iPLA_2_)	chr22:38,507,502–38,601,697	No
PTGS1	Prostaglandin-endoperoxide Synthase 1 (COX-1)	chr9:125,132,824–125,157,982	No
PTGS2	Prostaglandin-endoperoxide synthase 2 (COX-2)	chr1:186,640,923–186,649,559	No
PTGDS	Prostaglandin D2 Synthase	chr9:139,871,956–139,879,887	No
PTGES2	Prostaglandin E Synthase 2	chr9:130,882,972–130,890,741	No
PEGER2	Prostaglandin E Receptor 2	chr14:52,781,016–52,795,324	No
PTGER4	Prostaglandin E Receptor 4	chr5:40,679,600–40,693,837	No
PPARG	Peroxisome Proliferator Activated Receptor Gamma (PPAR-*y*)	chr3:12,328,867–12,475,855	No

## Conclusion

This review shows altered cellular pathology behind a diminished flush response. First, diminished flush is not only caused by vasodilators, but also by altered protein expression, protein activity, and inflammatory imbalance. Altered protein levels in the GPR109A-COX-prostaglandin pathways include membrane phospholipids, GPR109A, enzymes, cPLA_2_ and COX-2, and prostaglandins with their receptors and downstream products, such as PGD_2_, PGE_2_, DP_1_, EP_2_, EP_4_, 15d-PGJ_2_, and PPAR-*y*. Furthermore, we found that there was an inflammatory imbalance in the flush response. Although there is a possibility of genetic alteration in GPR109A, it is possible that environmental factors, such as oxidative stress, may alter receptor conformation, causing reduced receptor-ligand bonds, resulting in diminished flush. Second, as patient demographics interfere with the flush effect, future studies should consider the age, illness stage, ethnicity, use of antipsychotics, and presence of health comorbidities in their participants. The niacin skin flush test is essentially used to diagnose patients at their prodromal stage; however, this review contains limited research on the altered cell pathology at the prodromal stage. This review well supports the evidence for M1 microglia activation; however, evidence on neurons is weak, as there is no direct evidence linking diminished flush response to neurons. Given that microglia and neurons share a bidirectional relationship, it is likely that M1 activation may indirectly influence neuronal apoptosis. Lastly, JNK inhibition can inhibit M1 activation, neuronal apoptosis, and reduce inflammatory mediators, NF-κB, IL-1B, and TNF-α, and influence protein phosphorylation or expression, cPLA_2_, COX-2, and PPAR-*y*, respectively. Although further investigation is required to understand whether ROS-mediated JNK may influence GPR109A, we believe that the ability of JNK to control multiple targets in the diminished flush response would make it a good therapeutic target for schizophrenia. Future research should investigate whether stimulation of GPR109A results in PGD_2_ or PGE_2_ release from microglial cells and whether this is mediated by the JNK pathway. Future research should also bear in mind that Table 1 has established a match with 128 GWAS, which may be essential for the updated GWAS for schizophrenia in the future.

## Author Contributions

The author confirms being the sole contributor of this work and has approved it for publication.

## Acknowledgements

I would like to thank my Supervisor, Dr. Brian Morris for his support and guidance which has enabled me to complete this review.

## Conflict of Interest

The author declares that the research was conducted in the absence of any commercial or financial relationships that could be construed as a potential conflict of interest.

## Publisher's Note

All claims expressed in this article are solely those of the authors and do not necessarily represent those of their affiliated organizations, or those of the publisher, the editors and the reviewers. Any product that may be evaluated in this article, or claim that may be made by its manufacturer, is not guaranteed or endorsed by the publisher.
