# Supplementary material for: Inflammation and JNK's Role in Niacin-GPR109A Diminished Flushed Effect in Microglial and Neuronal Cells With Relevance to Schizophrenia
Source: Front Psychiatry. 2021 Nov 30;12:771144. doi: 10.3389/fpsyt.2021.771144 (PMC8668869; doi:10.3389/fpsyt.2021.771144)
Supplement: Supplementary file 1 [file Presentation_1.PPTX]

## Slide 1
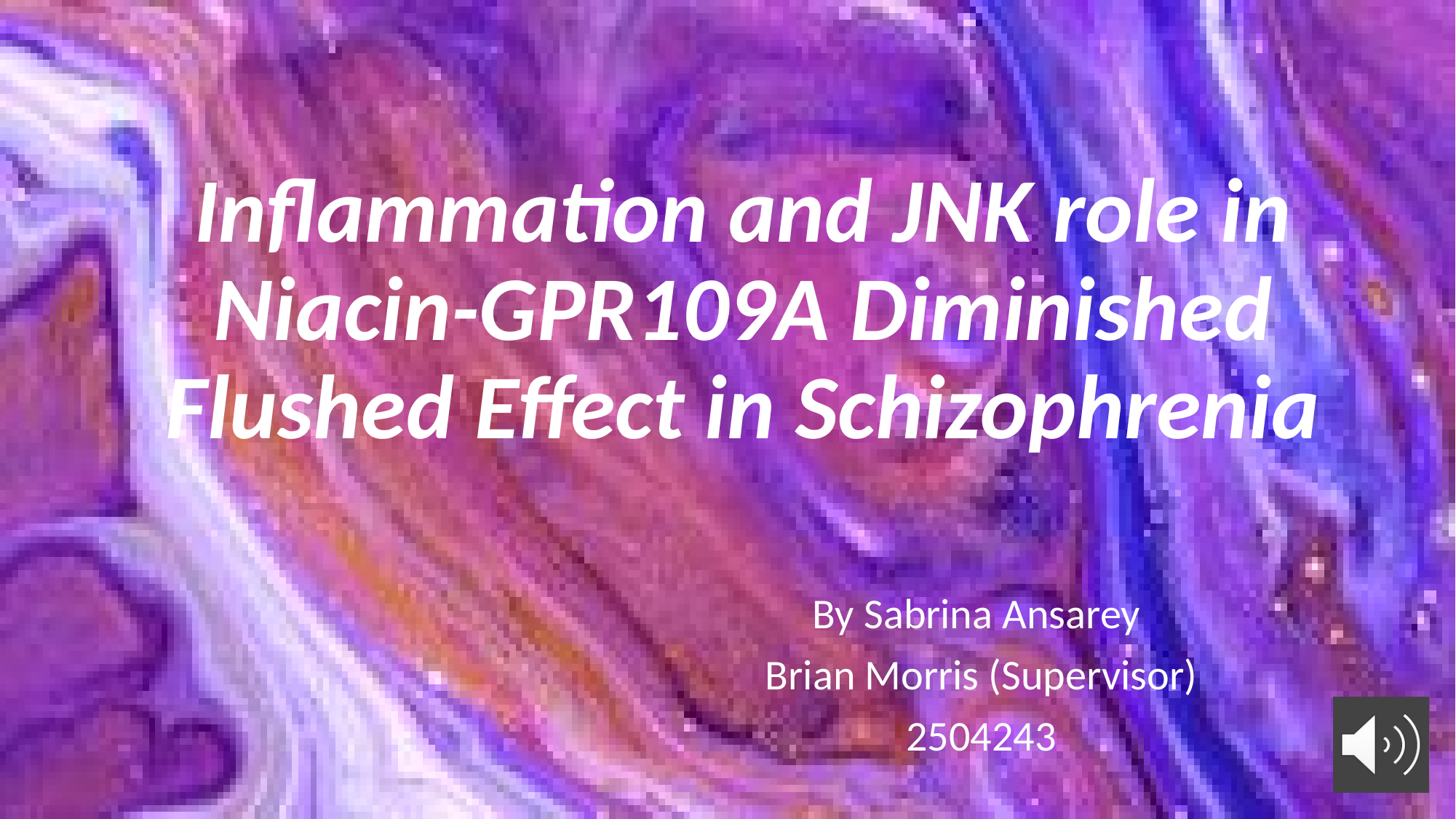

# Inflammation and JNK role in Niacin-GPR109A Diminished Flushed Effect in Schizophrenia
By Sabrina Ansarey
Brian Morris (Supervisor)
2504243

## Slide 2
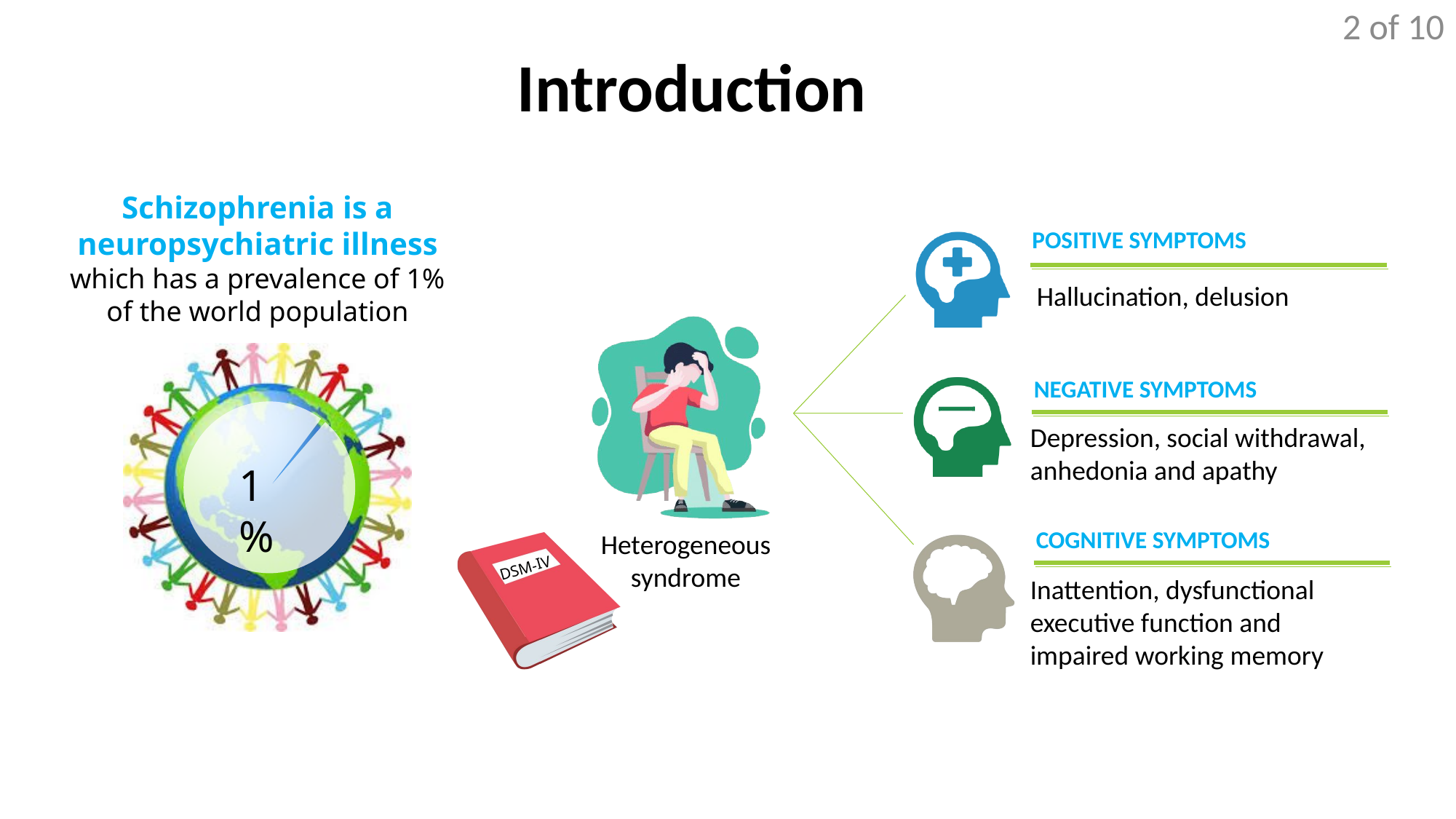

2 of 10
# Introduction
Schizophrenia is a neuropsychiatric illness which has a prevalence of 1% of the world population
POSITIVE SYMPTOMS
Hallucination, delusion
### Chart
| Category | Sales |
|---|---|
| 1st Qtr | 1.0 |
| 2nd Qtr | 99.0 |NEGATIVE SYMPTOMS
Depression, social withdrawal, anhedonia and apathy
1%
DSM-IV
COGNITIVE SYMPTOMS
Heterogeneous syndrome
Inattention, dysfunctional executive function and impaired working memory

## Slide 3
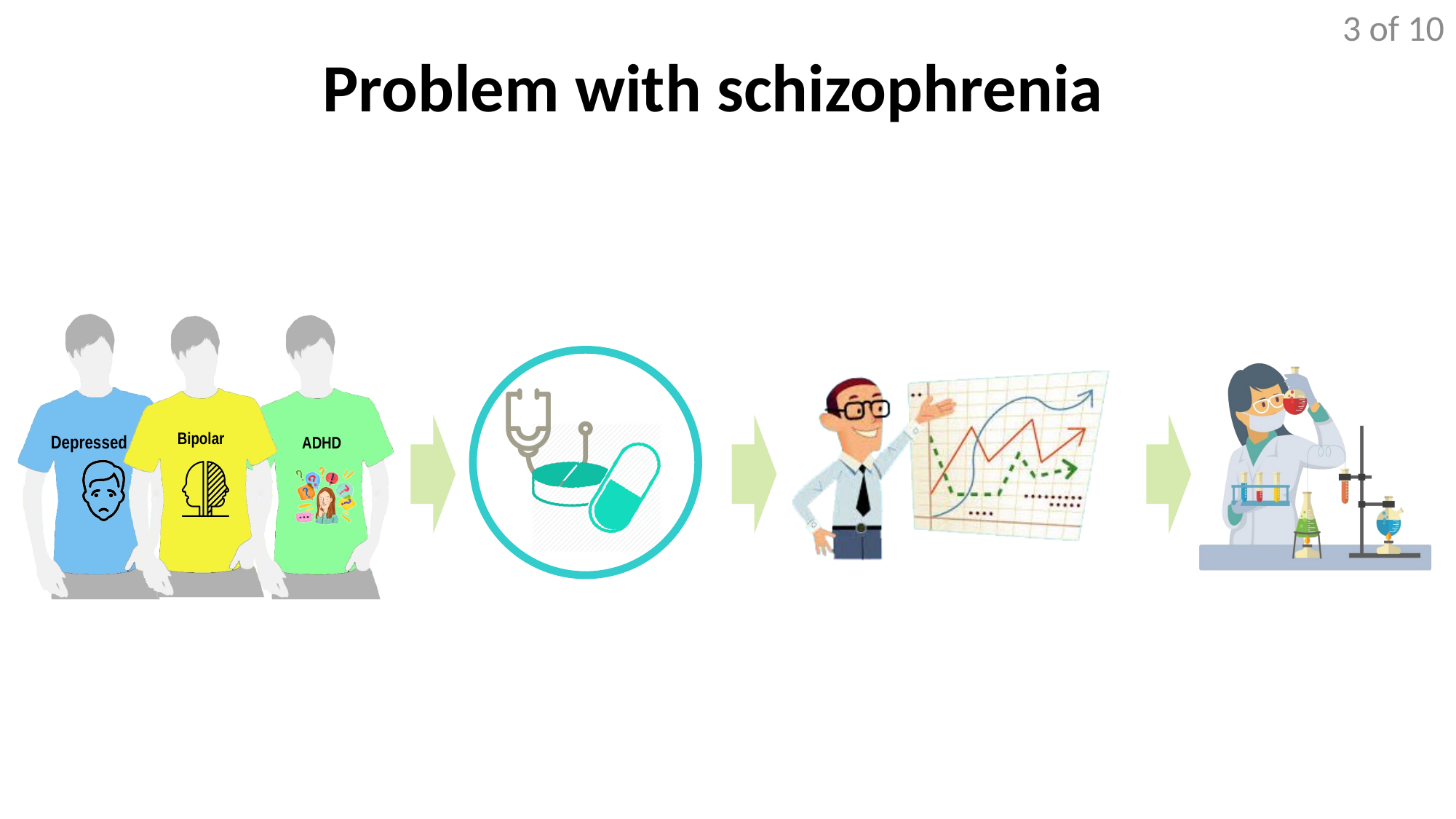

3 of 10
# Problem with schizophrenia
ADHD
Bipolar
Depressed

## Slide 4
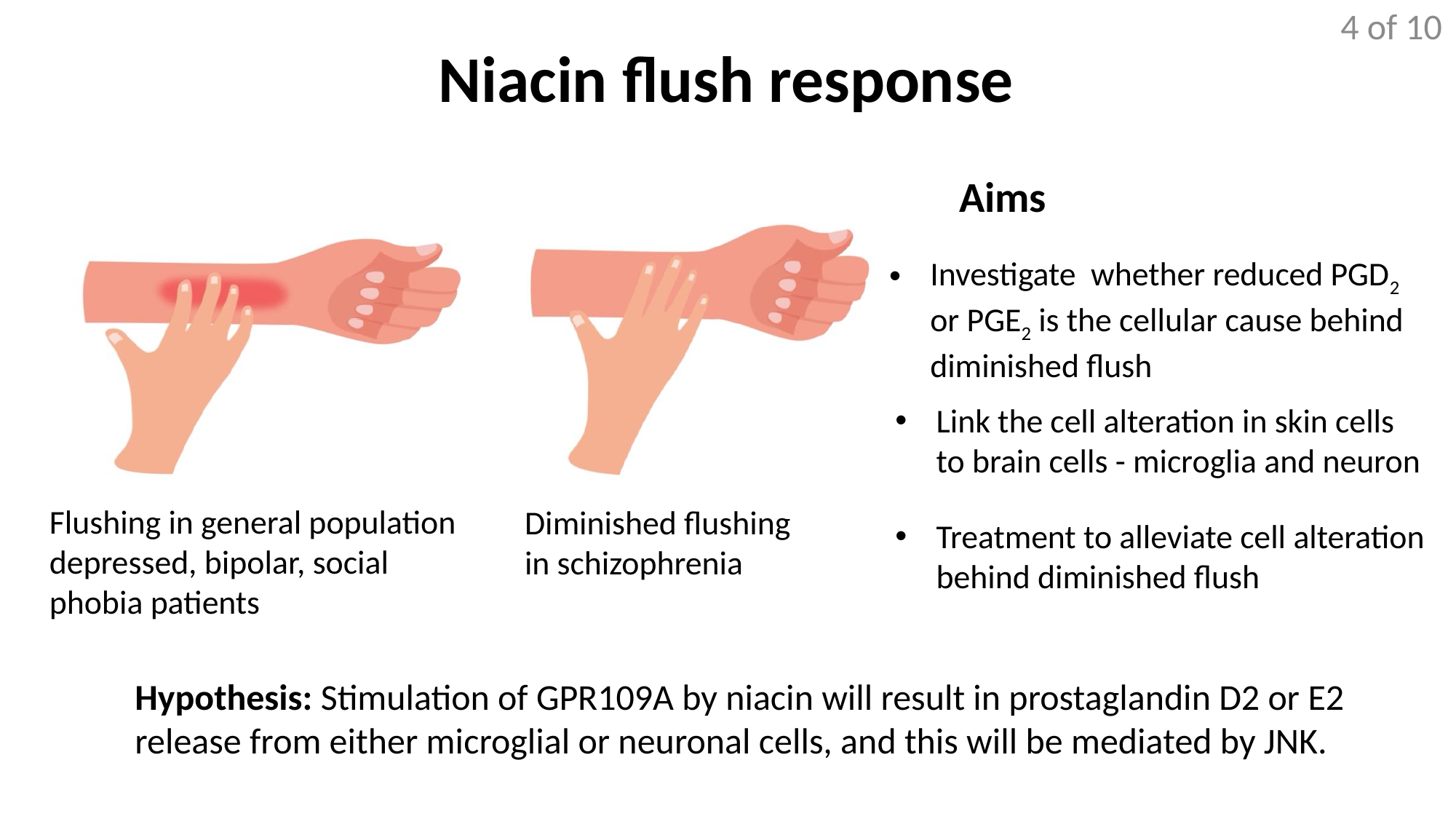

4 of 10
# Niacin flush response
Aims
Investigate whether reduced PGD2
or PGE2 is the cellular cause behind diminished flush
Link the cell alteration in skin cells
to brain cells - microglia and neuron
Treatment to alleviate cell alteration behind diminished flush
Flushing in general population depressed, bipolar, social phobia patients
Diminished flushing in schizophrenia
Hypothesis: Stimulation of GPR109A by niacin will result in prostaglandin D2 or E2 release from either microglial or neuronal cells, and this will be mediated by JNK.

## Slide 5
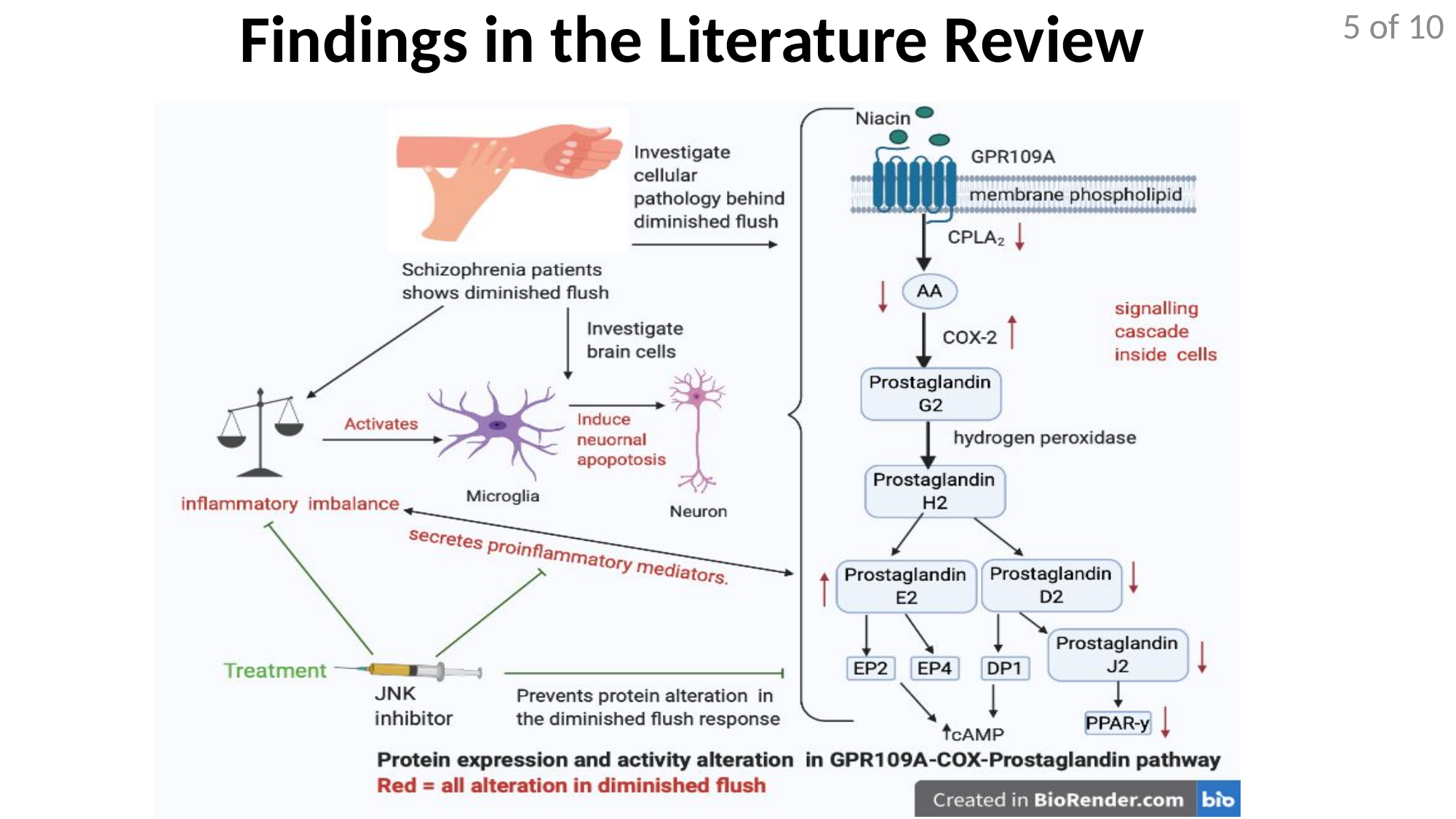

Findings in the Literature Review
5 of 10

## Slide 6
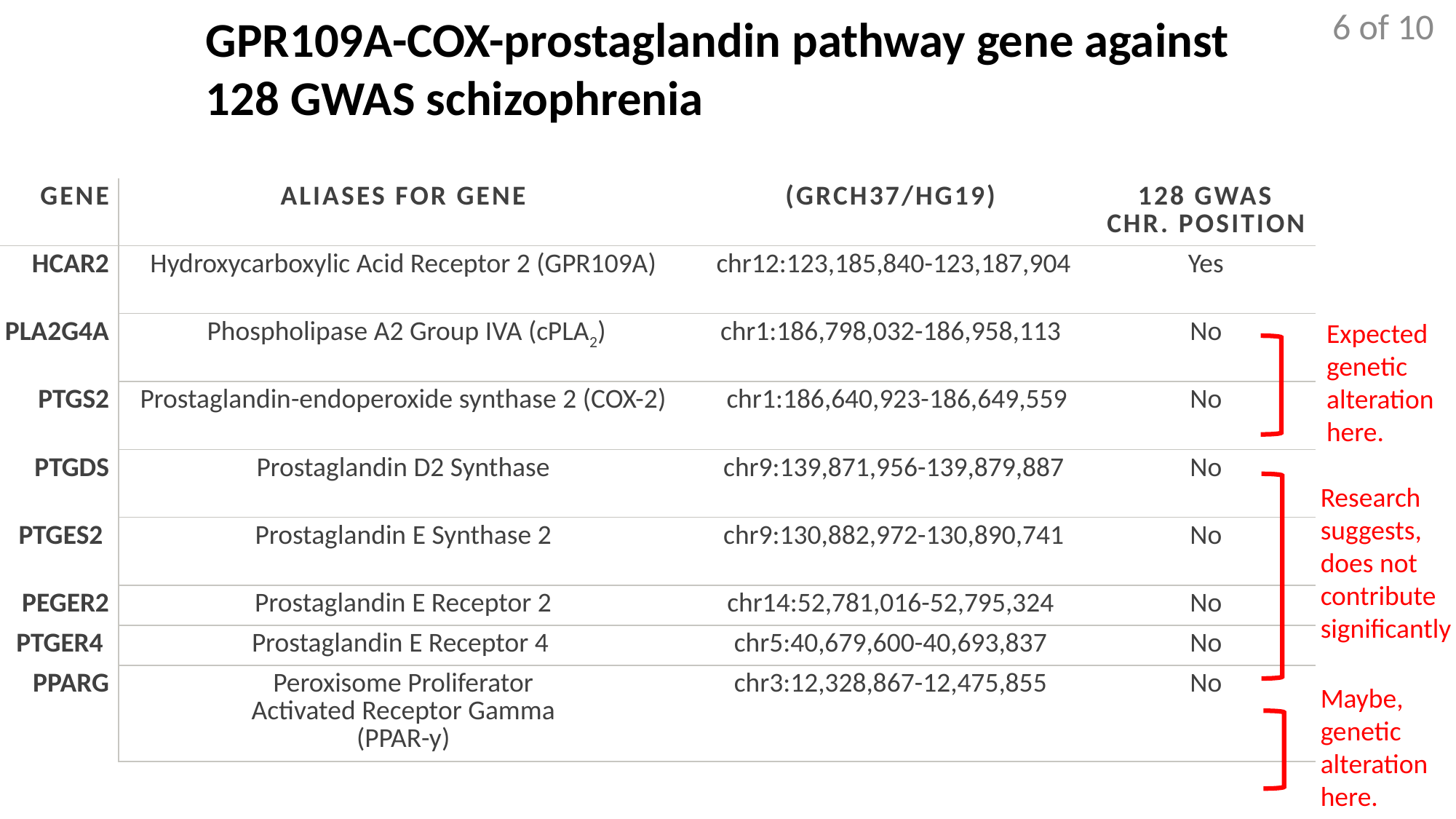

GPR109A-COX-prostaglandin pathway gene against 128 GWAS schizophrenia
6 of 10
| Gene | Aliases for Gene | (GRCh37/hg19) | 128 GWAS Chr. Position |
| --- | --- | --- | --- |
| HCAR2 | Hydroxycarboxylic Acid Receptor 2 (GPR109A) | chr12:123,185,840-123,187,904 | Yes |
| PLA2G4A | Phospholipase A2 Group IVA (cPLA2) | chr1:186,798,032-186,958,113 | No |
| PTGS2 | Prostaglandin-endoperoxide synthase 2 (COX-2) | chr1:186,640,923-186,649,559 | No |
| PTGDS | Prostaglandin D2 Synthase | chr9:139,871,956-139,879,887 | No |
| PTGES2 | Prostaglandin E Synthase 2 | chr9:130,882,972-130,890,741 | No |
| PEGER2 | Prostaglandin E Receptor 2 | chr14:52,781,016-52,795,324 | No |
| PTGER4 | Prostaglandin E Receptor 4 | chr5:40,679,600-40,693,837 | No |
| PPARG | Peroxisome Proliferator Activated Receptor Gamma (PPAR-y) | chr3:12,328,867-12,475,855 | No |
Expected genetic alteration here.
Research suggests, does not contribute significantly
Maybe, genetic alteration here.

## Slide 7
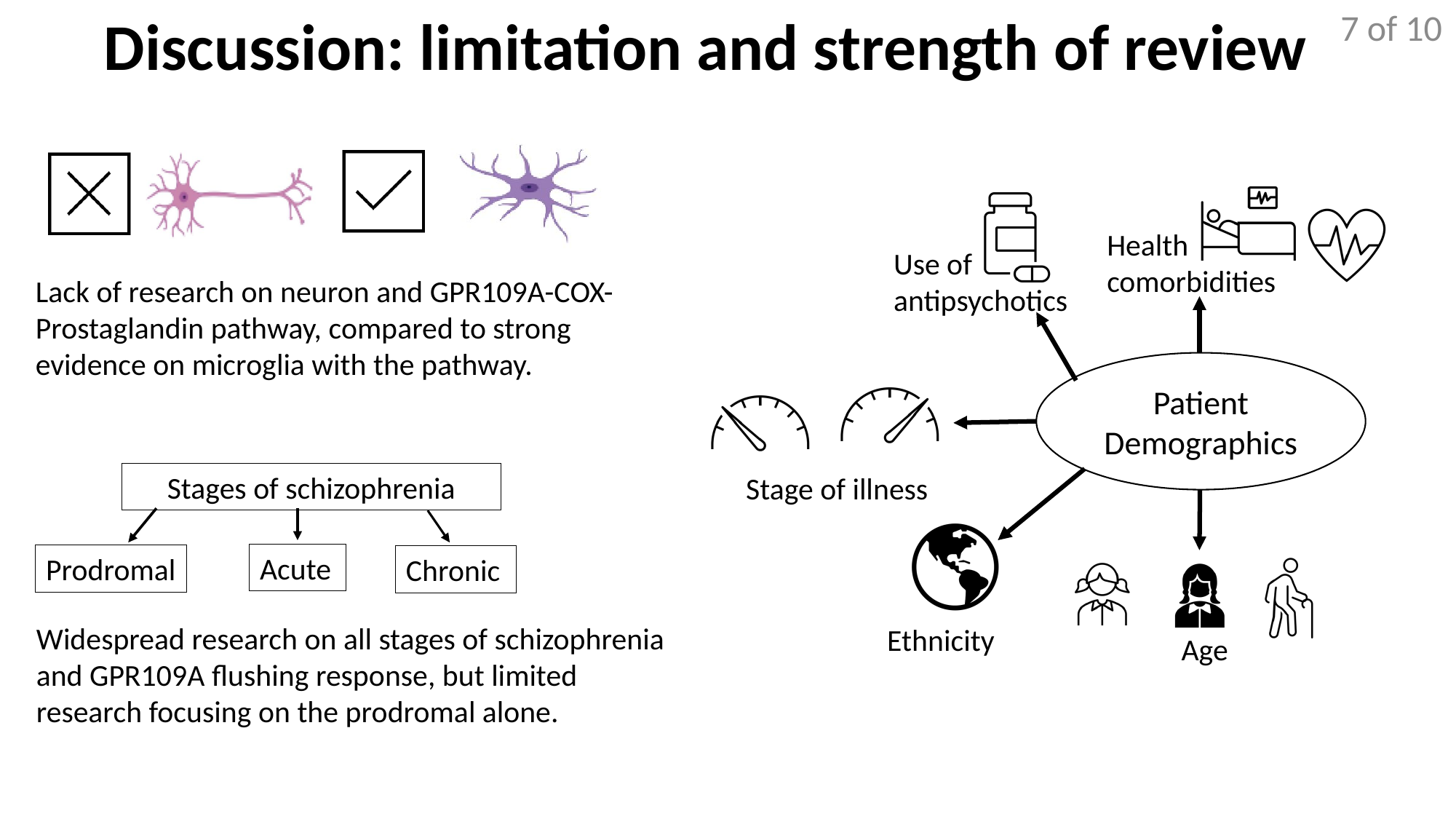

# Discussion: limitation and strength of review
7 of 10
Lack of research on neuron and GPR109A-COX-Prostaglandin pathway, compared to strong evidence on microglia with the pathway.
Health comorbidities
Use of antipsychotics
Patient Demographics
Stage of illness
Ethnicity
Age
Stages of schizophrenia
Acute
Prodromal
Chronic
Widespread research on all stages of schizophrenia and GPR109A flushing response, but limited research focusing on the prodromal alone.

## Slide 8
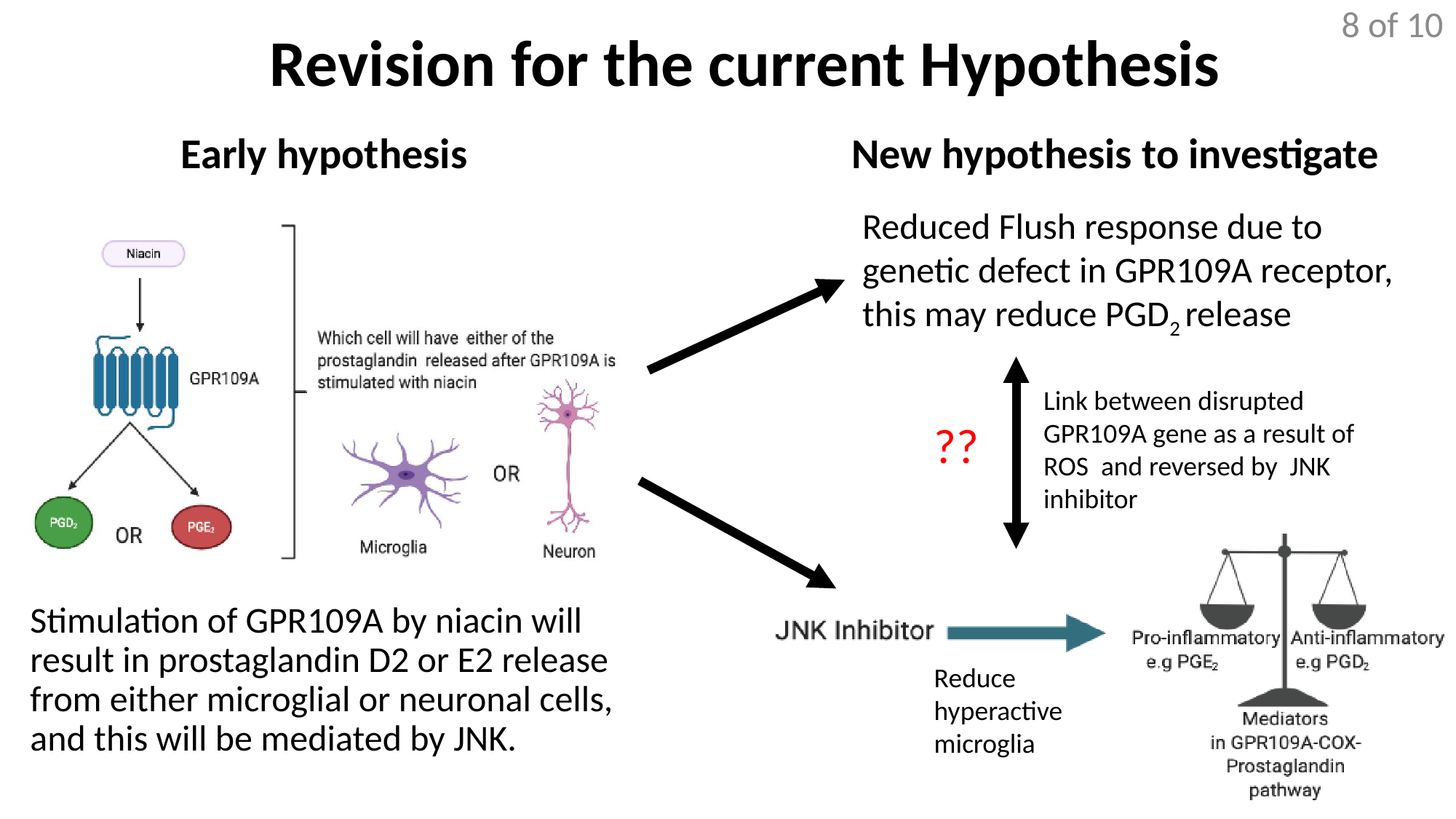

8 of 10
# Revision for the current Hypothesis
Early hypothesis
New hypothesis to investigate
Reduced Flush response due to genetic defect in GPR109A receptor, this may reduce PGD2 release
Link between disrupted GPR109A gene as a result of ROS and reversed by JNK inhibitor
??
Reduce hyperactive microglia
Stimulation of GPR109A by niacin will result in prostaglandin D2 or E2 release from either microglial or neuronal cells, and this will be mediated by JNK.

## Slide 9
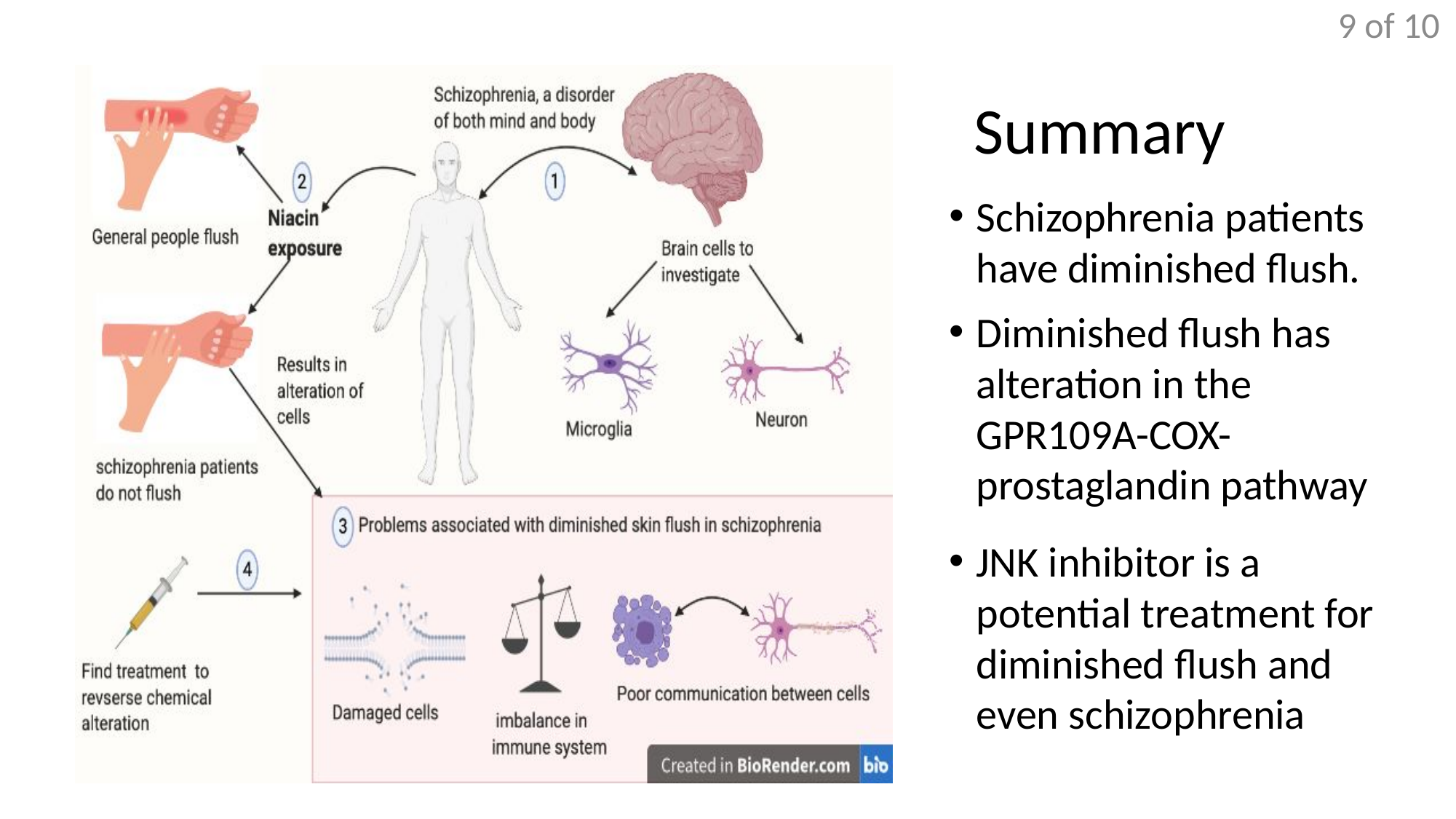

9 of 10
# Summary
Schizophrenia patients have diminished flush.
Diminished flush has alteration in the GPR109A-COX-prostaglandin pathway
JNK inhibitor is a potential treatment for diminished flush and even schizophrenia

## Slide 10
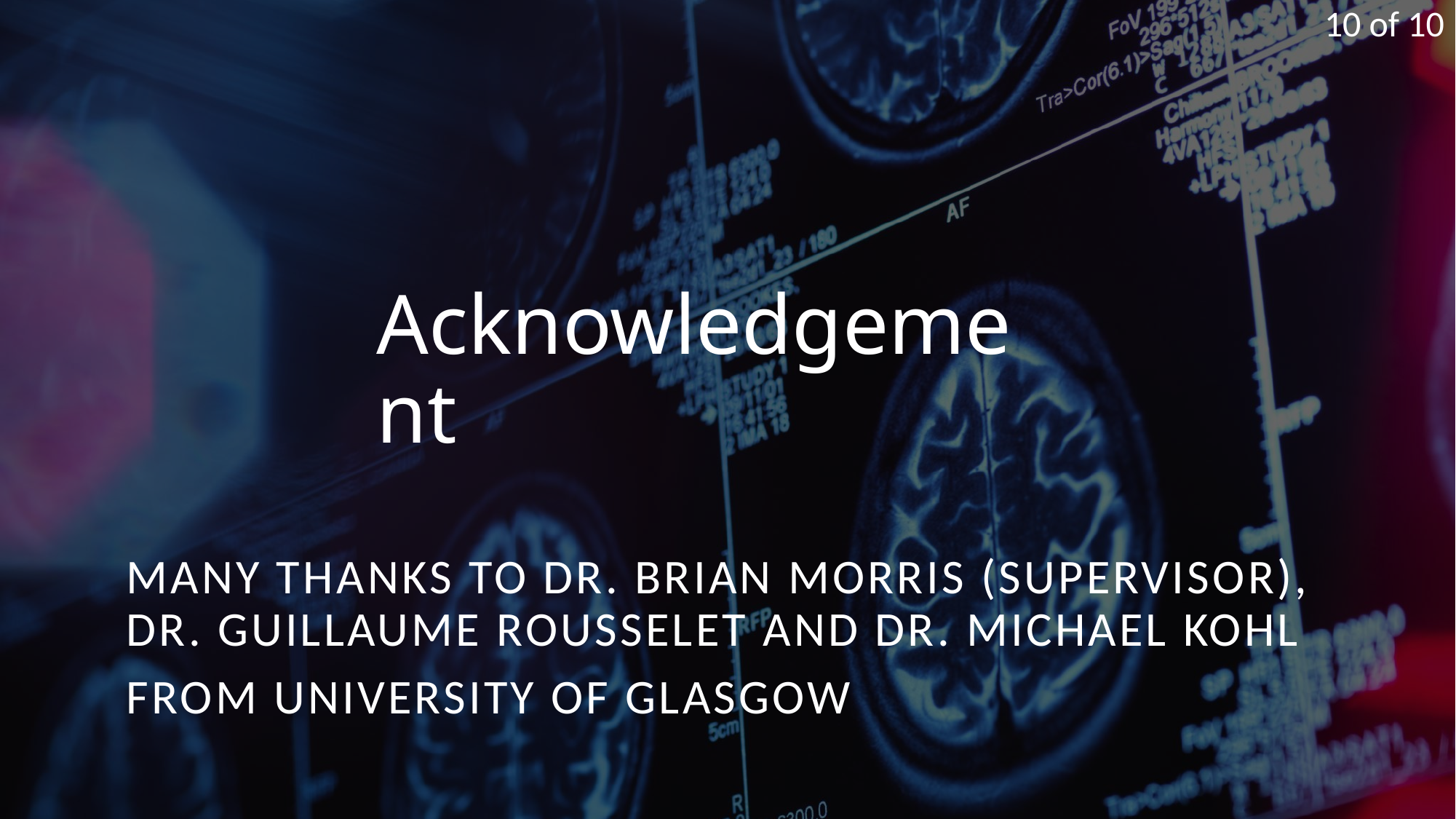

10 of 10
# Acknowledgement
Many thanks to Dr. Brian Morris (supervisor),
Dr. Guillaume Rousselet and Dr. Michael Kohl
From University of Glasgow
